# Hemizygous *Le-Cre* Transgenic Mice Have Severe Eye Abnormalities on Some Genetic Backgrounds in the Absence of *LoxP* Sites

**DOI:** 10.1371/journal.pone.0109193

**Published:** 2014-10-01

**Authors:** Natalie J. Dorà, J. Martin Collinson, Robert E. Hill, John D. West

**Affiliations:** 1 Centre for Integrative Physiology, University of Edinburgh, Edinburgh, United Kingdom; 2 Institute of Medical Sciences, University of Aberdeen, Aberdeen, United Kingdom; 3 Medical Research Council Human Genetics Unit, Medical Research Council Institute of Genetics and Molecular Medicine, University of Edinburgh, Edinburgh, United Kingdom; Childrens Hospital Los Angeles, United States of America

## Abstract

Eye phenotypes were investigated in *Le-Cre^Tg/−^*; *Pax6^fl/+^* mice, which were expected to show tissue-specific reduction of Pax6 in surface ectoderm derivatives. To provide a better comparison with our previous studies of *Pax6^+/−^* eye phenotypes, hemizygous *Le-Cre^Tg/−^* and heterozygous *Pax6^fl/+^*mice were crossed onto the CBA/Ca genetic background. After the *Le-Cre* transgene had been backcrossed to CBA/Ca for seven generations, significant eye abnormalities occurred in some hemizygous *Le-Cre^Tg/−^*; *Pax6^+/+^* controls (without a floxed *Pax6^fl^* allele) as well as experimental *Le-Cre^Tg/−^*; *Pax6^fl/+^* mice. However, no abnormalities were seen in *Le-Cre^−/−^*; *Pax6^fl/+^* or *Le-Cre^−/−^*; *Pax6^+/+^* controls (without the *Le-Cre* transgene). The severity and frequency of the eye abnormalities in *Le-Cre^Tg/−^*; *Pax6^+/+^* control mice diminished after backcrossing *Le-Cre^Tg/−^* mice to the original FVB/N strain for two generations, showing that the effect was reversible. This genetic background effect suggests that the eye abnormalities are a consequence of an interaction between the *Le-Cre* transgene and alleles of unknown modifier genes present in certain genetic backgrounds. The abnormalities were also ameliorated by introducing additional Pax6 gene copies on a CBA/Ca background, suggesting involvement of Pax6 depletion in *Le-Cre^Tg/−^*; *Pax6^+/+^* mice rather than direct action of Cre recombinase on cryptic pseudo-*loxP* sites. One possibility is that expression of Cre recombinase from the *Pax6-Le* regulatory sequences in the *Le-Cre* transgene depletes cofactors required for endogenous *Pax6* gene expression. Our observation that eye abnormalities can occur in hemizygous *Le-Cre^Tg/−^*; *Pax6^+/+^* mice, in the absence of a floxed allele, demonstrates the importance of including all the relevant genetic controls in Cre-*loxP* experiments.

## Introduction

*Tg(Pax6-cre,GFP)1Pgr* transgenic mice (hereafter abbreviated to *Le-Cre* transgenic mice) express Cre recombinase from *Pax6-Le* tissue-specific regulatory elements (the *Pax6* surface ectoderm enhancer and P0 promoter) in the pancreas and developing head surface ectoderm from embryonic day (E) 8.75 [1]. In their original study, Ashery-Padan *et al.* produced *Le-Cre^Tg/−^*; *Pax6^fl/lacZ^* mice, which were hemizygous for the *Le-Cre* transgene and carried both the *Pax6^lacZ^* null and floxed *Pax6^fl^* alleles [1]. Pax6 was deleted in the head surface ectoderm lineage early in development, demonstrating that, in the absence of Pax6, the lens fails to develop and the optic cup develops abnormally. Subsequently *Le-Cre* transgenic mice have been widely used to delete floxed alleles of other genes in the developing surface ectoderm including *Tfap2a* [2], *Fgfr2* [3], *Ctnnb* (previously *Catnb*; β-catenin) [4], *Six3* [5], *Klf4* [6], [7], *Klf5* [8], [9], *Pnn* [10], *Spry1* and *Spry2* [11], *Rac1* [12], *Ndst1* (in a *Ndst2^−/−^* background) [13], *Ilk* [14], *Vegfa* [15] and *Cited2* (either alone or in combination with floxed *Vegfa* or floxed *Hif1a*) [16], [17], [18].

We conditionally excised a single *Pax6* allele in the head surface ectoderm and derivatives (lens, conjunctiva and corneal epithelium) of *Le-Cre^Tg/−^*; *Pax6^fl/+^* mice to reduce Pax6 levels in these tissues rather than completely delete it. Pax6 levels are reduced globally in *Pax6^+/−^* mice, which are heterozygous for any *Pax6^−^* null allele (e.g. *Pax6^+/Sey^* or *Pax6^+/Sey-Neu^*). This global reduction results in a complex combination of abnormal phenotypes in the fetal and adult cornea, which disrupts corneal homeostasis [19], [20], [21], [22] and affects wound healing [23], [24]. We aimed to analyse the consequences of tissue-specific depletion of Pax6 on the corneal phenotype and compare this to the previously reported consequences of globally reducing Pax6 levels in *Pax6^+/−^* heterozygotes, thereby distinguishing abnormalities caused by low levels of Pax6 in the surface ectoderm lineage from those caused by low levels of Pax6 in other tissues, such as the optic cup. As the lens produces growth factors and probably influences the development of other anterior segment tissues [25], [26], we anticipated that depletion of Pax6 in the surface ectoderm tissues might also affect neighbouring tissues not derived from this lineage. This could occur if reduced Pax6 affected the production of signalling molecules or if Pax6 was itself secreted extracellularly, as reported for the developing chick nervous system [27].

Two previous studies used a similar approach and showed that reducing Pax6 levels in the surface ectoderm of *Le-Cre^Tg/−^*; *Pax6^fl/+^* eyes was sufficient to produce a number of developmental abnormalities that are characteristic of *Pax6^+/−^* heterozygotes. Some of these directly affected the lens and corneal epithelium (e.g. the lens was small and often remained attached to the cornea), suggesting an autonomous requirement for normal Pax6 gene dosage during development of these tissues [28]. However, other abnormalities suggested that normal Pax6 gene dosage in the surface ectoderm tissues is also required for normal development of some neighbouring tissues, implying a non-cell-autonomous mode of action. The trabecular meshwork and Schlemm's canal failed to develop and adhesions occurred between the peripheral iris and cornea causing the iridocorneal angle to be closed in the adult *Le-Cre^Tg/−^*; *Pax6^fl/+^* eye [29]. These abnormalities suggest that reduced Pax6 gene dosage in the surface ectoderm tissues can cause abnormalities in the neighbouring ocular mesenchyme.

We planned to extend these experiments and use *Le-Cre^Tg/−^*; *Pax6^fl/+^* eyes with a panel of morphological, immunohistochemical and wound-healing endpoints to consider a wider range of abnormal corneal phenotypes, as previously reported for adult *Pax6^+/−^* heterozygotes. To allow direct comparisons with phenotypes in our previous studies of *Pax6^+/−^* mice on a predominantly CBA/Ca genetic background, we crossed the *Le-Cre* transgene onto this genetic background. After several generations, some hemizygous *Le-Cre^Tg/−^*; *Pax6^+/+^* control mice had eye abnormalities even though they did not carry a floxed *Pax6^fl^* allele. Although eye abnormalities have been observed in homozygous *Le-Cre^Tg/Tg^* mice on some genetic backgrounds, this has only been reported as a brief abstract [30] and we are not aware of any previous reports of eye abnormalities in hemizygous *Le-Cre^Tg/−^* mice. The unexpected control phenotype in our study was only identified because all three genetic controls (*Le-Cre^Tg/−^*; *Pax6^+/+^*, *Le-Cre^−/−^*; *Pax6^fl/+^* and *Le-Cre^−/−^*; *Pax6^+/+^*) were examined alongside experimental *Le-Cre^Tg/−^*; *Pax6^fl/+^* eyes throughout the experiment, as the genetic background changed.

We have characterised the eye defects in *Le-Cre^Tg/−^*; *Pax6^+/+^* mice so that others are aware of the genetic background issues with the *Le-Cre* transgene. It is possible that some previous reports of *Le-Cre*-mediated conditional gene deletions may have conflated the abnormal eye phenotypes, resulting from a deleted floxed gene, with unexpected abnormal phenotypes, arising from effects of the *Le-Cre* transgene itself.

## Materials and Methods

### Ethics Statement

All the animal work in this study was approved by the University of Edinburgh Ethical Review Committee (applications PL21–06 and PL26–11) and performed in accordance with UK Home Office regulations under project license numbers PPL 60/3635 and PPL 60/4302. Mice were killed by cervical dislocation following inhalation of gaseous isoflurane anaesthetic. The highest standards of animal care were maintained throughout the study.

### Experimental animals

**Figure 1:**
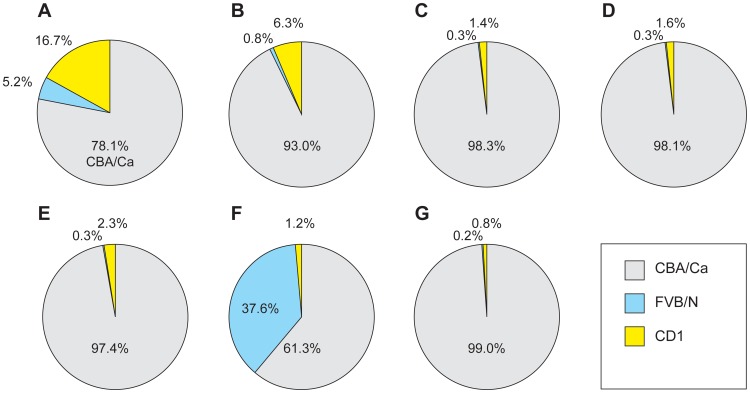
Genetic background of mice used at different stages of the study. (**A**) Adult mice from (*Le-Cre^Tg/−^*; *Pax6^+/+^* × *Le-Cre^−/−^*; *Pax6^fl/+^*) crosses in stage 1. (**B**) Adult mice from (*Le-Cre^Tg/−^*; *Pax6^+/+^* × *Le-Cre^−/−^*; *Pax6^fl/+^*) crosses used for wound healing experiment in stage 2. (**C**) Fetal and juvenile mice from (*Le-Cre^Tg/−^*; *Pax6^+/+^* × *Le-Cre^−/−^*; *Pax6^fl/+^*) crosses in stage 3. (**D**) Adult mice from (*Le-Cre^Tg/−^*; *Pax6^+/+^* × *Le-Cre^−/−^*; *Pax6^fl/+^*) crosses in stage 3. (**E**) Adult reporter mice from (*Le-Cre^Tg/−^* × *Z/AP*) crosses in stage 3. (**F**) Adult mice from (*Le-Cre^Tg/−^*; *Pax6^+/+^* × *Le-Cre^−/−^*; *Pax6^fl/+^*) crosses in stage 4. (**G**) Adult mice from (*Le-Cre^Tg/−^*; *Pax6^fl/+^* × *PAX77^Tg/−^*) crosses. See Table S1 and Materials and Methods for further details.

FVB/N mice were purchased from Charles River UK and other mice were bred and maintained in the Biomedical Research Facilities of the University of Edinburgh. Heterozygous *Pax6^tm1Ued/+^* (abbreviated to *Pax6^fl/+^*) mice [31] were obtained from Prof David Price (University of Edinburgh, UK) on an outbred CD1 genetic background. Hemizygous *Le-Cre^Tg/−^* mice (full name *Tg(Pax6-cre,GFP)1Pgr*; MGI number 3045749) [1] were obtained from Dr Ruth Ashery-Padan and Prof Peter Gruss (Max Plank Institute for Biophysical Chemistry, Goettingen, Germany) on an inbred FVB/N genetic background. (We use the genotype notation *Le-Cre^Tg/Tg^* to represent mice homozygous for the *Le-Cre* transgene, *Le-Cre^Tg/−^* for hemizygous mice and *Le-Cre^−/−^* for mice without the transgene.) We crossed both stocks to CBA/Ca to make the genetic background more consistent with our previous studies with *Pax6^+/−^* (*Pax6^+/Sey-Neu^*) mice. (Unlike CBA/J, FVB/N and some CD1 mice, the CBA/Ca inbred strain does not carry the *Pde6b^rd1^* retinal degeneration mutation.) *Le-Cre^Tg/−^*; *Pax6^+/+^*and *Le-Cre^−/−^*; *Pax6^fl/+^* mice were intercrossed to produce offspring of four genotypes, with or without the *Le-Cre* transgene and with or without the *Pax6^fl^* floxed allele of *Pax6*: (i) *Le-Cre^Tg/−^*; *Pax6^fl/+^*, (ii) *Le-Cre^Tg/−^*; *Pax6^+/+^*, (iii) *Le-Cre^−/−^*; *Pax6^fl/+^* and (iv) *Le-Cre^−/−^*; *Pax6^+/+^* (which is wild-type, WT). For the initial crosses (stage 1), the *Le-Cre^Tg/−^* mice used had been crossed to CBA/Ca for 3 or 4 generations (denoted as N3–N4) and the *Pax6^fl/+^* mice had been crossed to CBA/Ca for 1 or 2 generations. The average genetic background of the stage-1 progeny was estimated as approximately 78% CBA/Ca, 5% FVB and 17% CD1 (Table S1 and Fig. 1A). Wound healing studies were undertaken with stocks that had been backcrossed to CBA/Ca for a few more generations (*Le-Cre^Tg/−^* at N6; *Pax6^fl/+^* at N3). This is designated stage 2 and the average genetic background was estimated as approximately 93% CBA/Ca, 0.8% FVB and 6% CD1 (Table S1 and Fig. 1B). Further investigations of embryonic day (E)12.5 fetal stages to postnatal day (P) 10 and adults were undertaken in stage 3 after further backcrossing (*Le-Cre^Tg/−^* at N7-N8; *Pax6^fl/+^* at N5–N6). The average genetic background of the stage-3 progeny was estimated as approximately 98% CBA/Ca; 0.3% FVB and 1.5% CD1 (Table S1 and Figs. 1C,D). A final comparison (stage 4) was made using *Le-Cre^Tg/−^* mice that had been backcrossed to CBA/Ca for 8 generations and then crossed to FVB/N for 2 generations to change the genetic background and *Pax6^fl/+^* mice that had been crossed to CBA/Ca for 5 or 6 generations. The average genetic background of the stage-4 progeny was estimated as approximately 61% CBA/Ca, 38% FVB and 1.2% CD1 (Table S1 and Fig. 1F). Mice were genotyped by polymerase chain reaction (PCR) [1], [31]. The study was conducted continuously, *Le-Cre* mice used were all derived from a single backcross line and mice were bred from June 2010 to October 2012 as shown in Table S1. Experiments were all performed by the same person (NJD), using the same methods throughout the study.

Heterozygous *Pax6^+/Sey-Neu^* mice (abbreviated to *Pax6^+/−^*) were maintained by crossing them to inbred CBA/Ca mice and were considered congenic on that strain (>20 backcross generations). Heterozygotes were distinguished from wild-type littermates by eye size and genotypes were confirmed by PCR [32]. Mice carrying the *PAX77* transgene, comprising 5–7 copies of the human *PAX6* gene [33] were obtained from Prof Veronica van Heyningen and Dr Dirk A. Kleinjan (MRC Human Genetics Unit, Edinburgh) on an outbred CD1 genetic background. (We use the genotype notation *PAX77^Tg/Tg^* to represent mice homozygous for the *PAX77* transgene, *PAX77^Tg/−^* for hemizygous mice and *PAX77^−/−^* for mice without the transgene.) Hemizygous *PAX77^Tg/−^* mice used in this study had been backcrossed to the CBA/Ca genetic background for at least 20 generations (CBA/Ca-*PAX77^Tg/−^* congenic strain) and were identified by their small eye size with genotypes confirmed by PCR as described previously [34], [35]. The genetic background of *Le-Cre^Tg/−^*; *Pax6^fl/+^* mice used for crosses with CBA/Ca-*PAX77^Tg/−^* (*Le-Cre^Tg/−^* at N6; *Pax6^fl/+^* at N5) was approximately 98% CBA, 0.4% FVB and 1.6% CD1), which is equivalent to stage-3 mice, and the genetic background of mice produced by the *Le-Cre^Tg/−^*; *Pax6^fl/+^*×*PAX77^Tg/−^* cross was estimated as approximately 99% CBA/Ca, 0.2% FVB/N and 0.8% CD1 (Table S1 and Fig. 1G). Z/AP reporter mice express *lacZ* in all tissues but after recombination of *loxP* sites, *lacZ* is deleted and human placental alkaline phosphatase (AP) is expressed [36]. Z/AP mice were obtained on an outbred CD1 genetic background from Prof. David Price (University of Edinburgh, UK), crossed to CBA/Ca and genotyped, using PCR to detect the *lacZ* region of the Z/AP construct. The genetic background of mice produced by *Le-Cre^Tg^*×*Z/AP* crosses was estimated as approximately 97% CBA/Ca, 0.8% FVB/N and 2.3% CD1 (Table S1 and Fig. 1E) and is considered equivalent to the stage 3 genetic background.

Adult mice were killed by cervical dislocation, following inhalation of gaseous isoflurane anaesthetic, at 12 weeks and their eyes were removed immediately, weighed and fixed in 4% paraformaldehyde (PFA). For wound-healing studies, eyes were wounded *in situ* and removed for organ culture. For postnatal day (P) 2 and P10 samples, the day of birth was defined as P0 and samples were collected and fixed in 4% PFA. For embryonic stages, the morning that a vaginal plug was found was defined as embryonic day (E) 0.5; embryos were collected and heads were removed and fixed in 4% PFA. Only one eye per mouse (the right eye) was examined histologically or by immunohistochemistry so the numbers of eyes examined is also equal to the numbers of mice examined.

### Histology

Samples were fixed in 4% PFA overnight at 4°C, processed to wax and 7 µm sections cut. Adult eyes were cut in an anterior-posterior plane to include cornea, lens and retina. To avoid adult lenses shattering the wax block was kept wet during sectioning. Embryonic heads were cut anterior-posterior in a horizontal plane so sections through the developing eye included cornea, lens, and retina. Histological features were compared using standard haematoxylin and eosin (H & E) staining methods. For Periodic acid-Schiff's (PAS) staining, slides were washed in periodic acid for 15 minutes, rinsed in water and transferred to Schiff's reagent for 5 minutes.

### Morphometric measurements

Corneal diameters were measured using a stereomicroscope fitted with an eyepiece graticule. Tissue sections of adult eyes were viewed under a Zeiss Axioplan 2 compound microscope (x 40 objective) and captured images of the cornea measured using a calibrated Zeiss Axiovision 4.8 digital camera system. Sections from the central cornea were measured in six regions (2 peripheral, 2 intermediate and 2 central), mean thicknesses were calculated for peripheral, intermediate and central corneal epithelium. Cell layers were also counted for peripheral, intermediate and central regions in the same sections.

### Immunohistochemical staining

Sections were de-waxed in histoclear and re-hydrated through a graded alcohol series to water, incubated in 3% hydrogen peroxide in methanol for 20 minutes rehydrated in 70% ethanol and washed in phosphate buffered saline (PBS). Antigen unmasking was performed by incubating slides in 0.01 M citrate buffer (pH 6.0) in a water bath heated to 95°C for 35 minutes; slides were then allowed to cool for 20 min. and washed in PBS. Sections were treated with 10% blocking serum (species according to secondary antibody), 0.1% bovine serum albumin (BSA) in PBS for 1 hour at room temperature. Sections were incubated overnight at 4°C in the appropriate primary antibody, diluted in blocking serum, as follows. (The following primary antibodies were used. Pax6 staining: Developmental Studies Hybridoma Bank, University of Iowa diluted 1∶500. Cytokeratin 12 (K12): Santa Cruz Biotechnology sc-17101 diluted 1∶500. Cytokeratin 5 (K5): Abcam ab53121 diluted 1∶100. Cytokeratin 19 (K19): Lifespan Biosciences LS-C3372 diluted 1∶200). Slides were washed in PBS and incubated in blocking serum for 10 min. then incubated with secondary antibody, diluted in blocking serum, for 45 min. at room temperature. (The following secondary antibodies were used. Pax6 staining: Vector labs BA-9200 biotinylated goat anti-mouse diluted 1∶200. K12: Vector Labs BA-5000, biotinylated rabbit anti-goat IgG diluted 1∶200. K5 and K19: Vector Labs BA-1000, biotinylated goat anti-rabbit IgG diluted 1∶200). Slides were washed in PBS and incubated with avidin-biotin reagent (ABC RTU Vectastain, Vector Labs PK-7100). Antibody was then visualised by 3,3′-diaminobenzidine (DAB) stain (5.9 ml 20 mM Tris pH 7.6, 100 µl 50 mg/ml DAB, 1 µl H_2_O_2_) and slides were lightly counterstained with haematoxylin, dehydrated and coverslips were mounted with DPX mounting medium. Control slides were treated with blocking serum in place of primary antibody but otherwise treated identically.

### β-galactosidase and alkaline phosphatase histochemical staining on frozen sections

To prepare frozen tissue sections, samples were fixed in 4% PFA overnight, washed three times for 15 minutes in PBS and cryoprotected by treating with 15% sucrose in PBS for 1 hour at 4°C and then 30% sucrose overnight at 4°C. Tissues were incubated in optimal cutting temperature compound (OCT) at 4°C for 1 hour before they were embedded in OCT over dry ice. Blocks were stored at −80°C and warmed to −20°C before cryosections were cut at 10 µm, air dried for at least 1 hour and stored at −20°C.

Prior to staining, sections were fixed again with 0.2% gluteraldehyde in ice cold PBS for 10 min. For β-galactosidase (β-gal) staining, slides were washed three times for 5 min. in β-gal wash buffer (2 mM, 0.01% sodium deoxycholate, 0.02% Nonidet-P40 (NP-40) in 100 mM sodium phosphate, pH 7.3) and then incubated in β-gal stain (0.5 mg/ml X-gal, 5 mM potassium ferrocyanide, 5 mM potassium ferricyanide in β-gal wash buffer) for 4–6 h. at 37°C, while protected from light. Slides were rinsed in PBS, dehydrated through graded ethanols and coverslips were mounted with DPX mounting medium. For alkaline phosphatase (AP) staining, following fixation in 0.2% gluteraldehyde, slides were washed three times for 5 min. in PBS. Endogenous phosphatase activity was inactivated by incubating slides in PBS at 70–75°C for 30 min., slides were rinsed in PBS and washed in AP buffer (100 mM Tris-HCl, pH 9.5, 100 mM NaCl, 10 mM MgCl_2_) 10 min. Slides were incubated with nitro-blue tetrazolium and 5-bromo-4-chloro-3′-indolyphosphate (NBT/BCIP) stain for 10–30 min. at room temperature until the colour developed, washed in PBS, dehydrated and coverslips were mounted with DPX mounting medium.

### Corneal epithelial wound healing

Mice were killed and a wound was made in the central region of the cornea using a 1 mm diameter trephine blade, under a dissecting microscope while the eye was *in situ*. The area within the wound was debrided with an ophthalmological scalpel and the wound was visualized with fluorescein dye and photographed using a digital camera mounted on a dissecting microscope. Eyes were enucleated and placed in culture wells (cornea facing up) in corneal culture medium (CCM) [37] and kept in standard culture conditions for 24 h. The wound area was visualised and photographed at specific time points during culture and the wound diameter was measured using Adobe Photoshop CS v8.

### Statistical analysis

Left and right eyes were analysed separately because their genotypes are identical so they are not completely independent samples. Non-parametric Kruskal-Wallis tests and Dunn's multiple comparison post-hoc tests were performed using the statistics package GraphPad Prism 5.0c (GraphPad Software Inc., San Diego, USA). Fisher's Exact tests were calculated using an on-line statistics calculator (×http://vassarstats.net/odds2×2.html). The raw data are included in Supplementary Data S1.

## Results

### Morphology of *Le-Cre^Tg/-^; Pax6^fl/+^* eyes from stage-1 crosses

**Figure 2:**
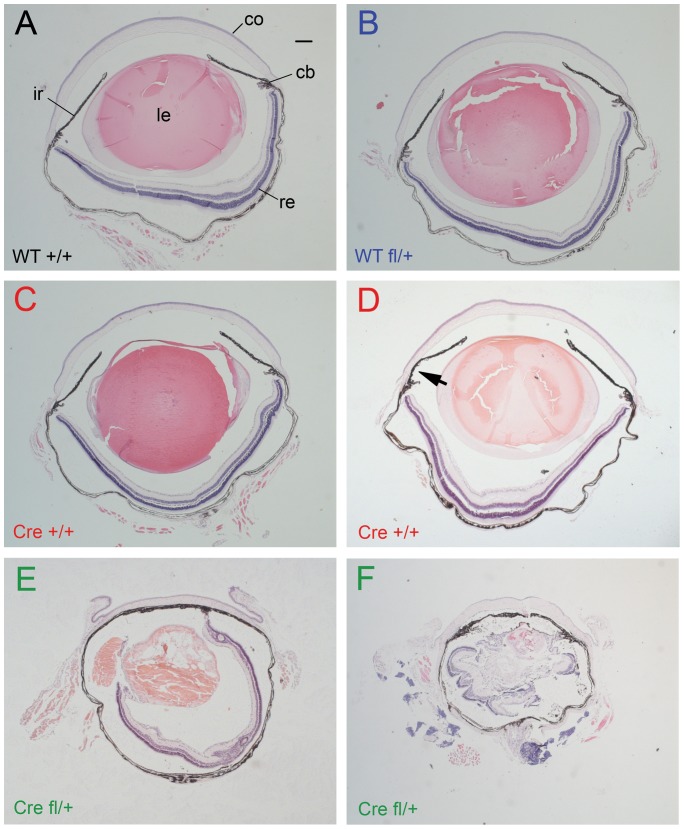
Histology of adult eyes of four genotypes from *Le-Cre^Tg/−^*; *Pax6^+/+^* × *Le-Cre^−/−^*; *Pax6^fl/+^* crosses in stage 1. H & E stained sections showing (**A–C**) normal morphology of (A) *Le-Cre^−/−^; Pax6^+/+^*, (B) *Le-Cre^−/−^; Pax6^fl/+^* and (C) *Le-Cre^Tg/−^; Pax6^+/+^* control eyes. (**D**) Another *Le-Cre^Tg/−^; Pax6^+/+^* eye with a closed irido-corneal angle (arrow) on the left of the photograph but an open angle to the right. (**E,F**) Experimental *Le-Cre^Tg/−^; Pax6^fl/+^*eyes showing typical abnormalities. Scale bar  =  200 µm. *Abbreviations*: cb, ciliary body; co, cornea; ir, iris; le, lens; re, retina. WT +/+ is *Le-Cre^−/−^*; *Pax6^+/+^*; WT fl/+ is *Le-Cre^−/−^*; *Pax6^fl/+^*; Cre +/+ is *Le-Cre^Tg/−^*; *Pax6^+/+^* and Cre fl/+ is *Le-Cre^Tg/−^*; *Pax6^fl/+^*. In each figure, the panel letters are colour coded to help distinguish the genotypes.

**Figure 3:**
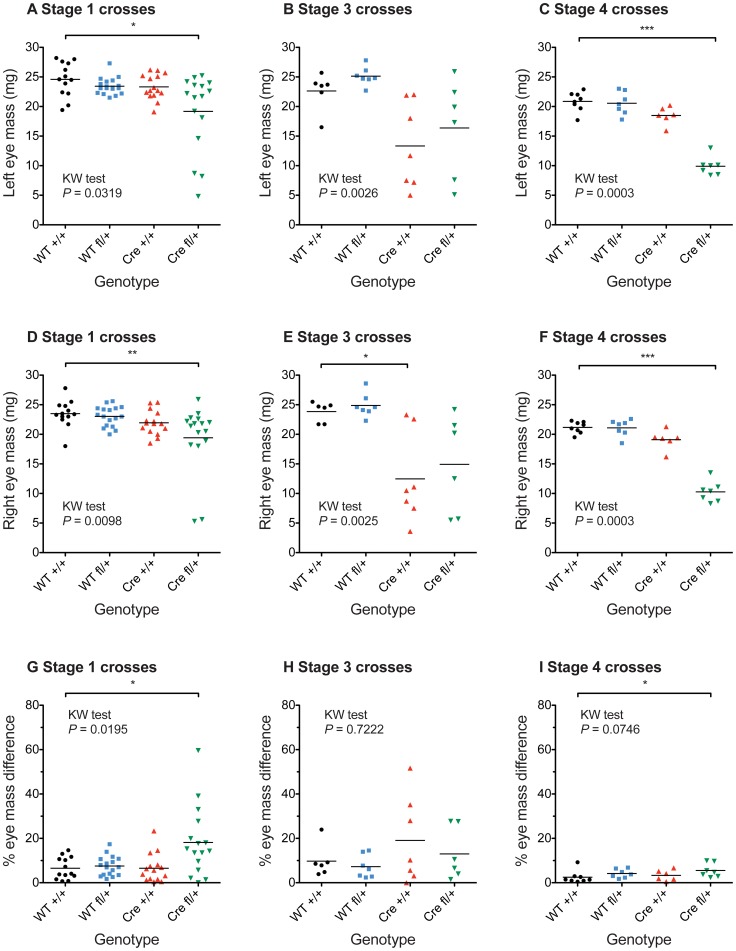
Variation in eye mass for different genotypes at different stages of the study. (**A–F**) Mass of left (A–C) and right (D–F) eyes of 12-week old mice from *Le-Cre^Tg/−^*; *Pax6^+/+^* × *Le-Cre^−/−^*; *Pax6^fl/+^* crosses on different genetic backgrounds: (A,D) stage 1 crosses (B,E) stage 3 crosses (C,F) stage 4 crosses. (**G–I**) The percentage eye mass difference, calculated for each mouse as (larger eye mass - smaller eye mass) ×100/(larger eye mass). *Abbreviations:* WT +/+ is *Le-Cre^−/−^*; *Pax6^+/+^*; WT fl/+ is *Le-Cre^−/−^*; *Pax6^fl/+^*; Cre +/+ is *Le-Cre^Tg/−^*; *Pax6^+/+^* and Cre fl/+ is *Le-Cre^Tg/−^*; *Pax6^fl/+^*. Results for all four genotypes were compared by non-parametric Kruskal-Wallis (KW) tests separately for each stage of the study (*P*-values are shown in the figure) and results for WT fl/+, Cre +/+ and Cre fl/+ were compared to WT +/+ by Dunn's multiple comparison post-hoc test: *P<0.05; **P<0.01; ***P<0.001.

**Figure 4:**
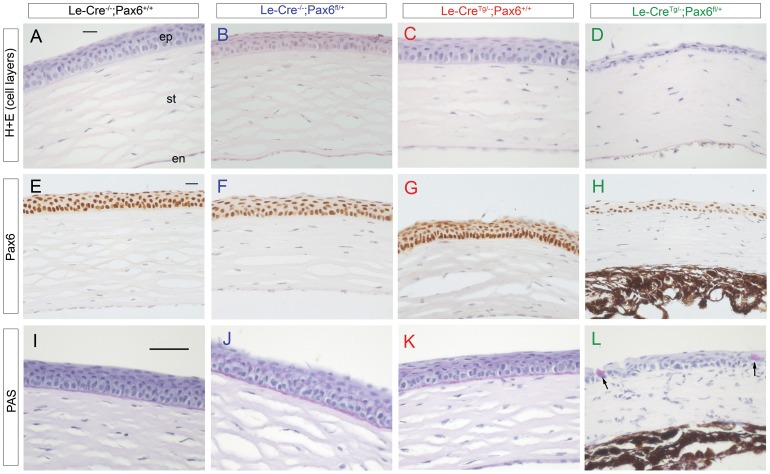
Histology and immunohistochemistry of adult corneas of four genotypes from *Le-Cre^Tg/−^*; *Pax6^+/+^* × *Le-Cre^−/−^*; *Pax6^fl/+^* crosses in stage 1. (**A–D**) H & E stained corneas showing normal morphology in controls (A–C) but reduced numbers of corneal epithelial layers in the *Le-Cre^Tg/−^; Pax6^fl/+^* cornea shown in (D). (**E–H**) Pax6 immunostaining (brown endpoint) in the corneal epithelium of all four genotypes. (**I–L**) Periodic acid-Schiff (PAS) staining (purple-magenta endpoint) in cornea showed goblet cells were absent from the corneal epithelium of the three control genotypes (I–K) and some experimental *Le-Cre^Tg/−^; Pax6^fl/+^* eyes (not shown) but present in other *Le-Cre^Tg/−^; Pax6^fl/+^* eyes (arrows in L). Scale bars: A–H = 20 µm, I–L = 50 µm. *Abbreviations*: en, corneal endothelium; ep, corneal epithelium; st, corneal stroma.

**Figure 5:**
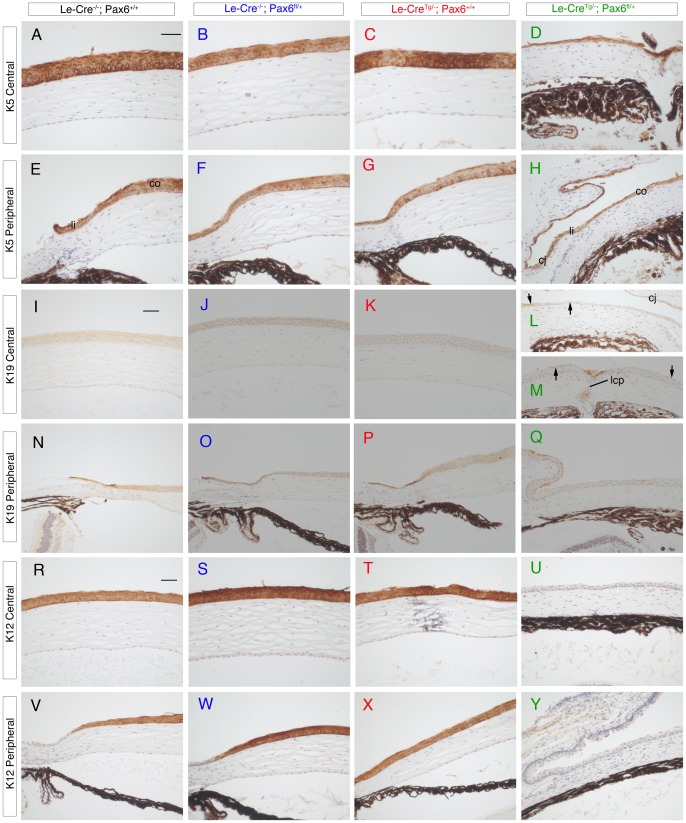
Immunohistochemistry for keratins 5, 19 and 12 in epithelia of the adult ocular surface of four genotypes from *Le-Cre^Tg/−^*; *Pax6^+/+^* × *Le-Cre^−/−^*; *Pax6^fl/+^* crosses in stage 1. (**A–H**) Keratin 5 (K5) immunostaining (brown endpoint) shows K5 is present in the central (A-D) and peripheral (E–H) corneal epithelium of all four groups. (**I–Q**) K19 immunostaining (brown endpoint) in the central corneal epithelium (I–M) shows K19 is absent in the three control groups (I–K) but patchy staining (arrows) is present in the *Le-Cre^−/−^; Pax6^fl/+^* central corneal epithelium (L,M). The cornea shown in M has a lens-corneal plug. In the peripheral corneal epithelium (N–Q), K19 is present in some cells of the limbus (and conjunctiva) in all four groups. (**R–X**) K12 immunostaining (brown endpoint) in the central corneal epithelium (R–U) and peripheral corneal and limbal epithelium (V–Y) shows K12 is absent from the *Le-Cre^Tg/−^; Pax6^fl/+^* ocular surface epithelium (U,Y) but present in the corneal epithelium of all three control groups (R–T and V–X). Scale bars  = 50 µm. *Abbreviations*: co, cornea; cj, conjunctiva; lcp, lens-corneal plug; li, limbus.

**Figure 6:**
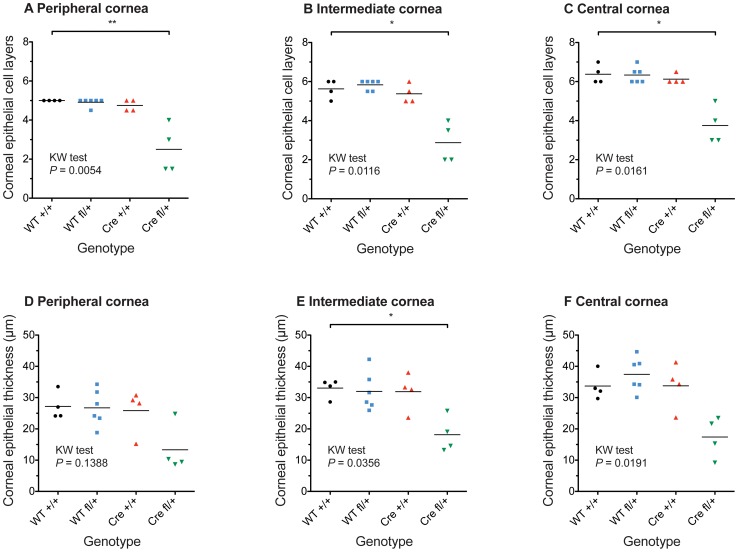
Variation in corneal epithelial thickness in different genotypes from stage-1 crosses. Variation in corneal thickness in 12-week-old mice of different genotypes from *Le-Cre^Tg/−^*; *Pax6^+/+^* × *Le-Cre^−/−^*; *Pax6^fl/+^* stage 1 crosses. Variation in (**A–C**) number of corneal epithelial layers, (**D–F**) corneal epithelial thickness (µm) at the periphery (A,D), intermediate (B,E) and central (C,F) cornea in 12 week old mice of different genotypes from *Le-Cre^Tg/−^*; *Pax6^+/+^* × *Le-Cre^−/−^*; *Pax6^fl/+^* stage 1 crosses. Results are means of two measurements at the periphery, intermediate and central regions of 4–6 eyes (1 eye per mouse) per genotype. *Abbreviations:* WT +/+ is *Le-Cre^−/−^*; *Pax6^+/+^*, WT fl/+ is *Le-Cre^−/−^*; *Pax6^fl/+^*, Cre +/+ is *Le-Cre^Tg/−^*; *Pax6^+/+^* and Cre fl/+ is *Le-Cre^Tg/−^*; *Pax6^fl/+^*. Results for all four genotypes were compared by non-parametric Kruskal-Wallis (KW) tests separately for each corneal region (*P*-values are shown in the figure) and results for WT fl/+, Cre +/+ and Cre fl/+ were compared to WT +/+ by Dunn's multiple comparison post-hoc test: * *P*<0.05;** *P*<0.01.

Eyes from the experimental and three control genotypes produced by *Le-Cre^Tg/−^*; *Pax6^+/+^*×*Le-Cre^−/−^*; *Pax6^fl/+^* crosses were compared in four discrete stages as the genetic background changed, as explained in the Materials and Methods section. The average genetic background of the stage-1 progeny was approximately 78% CBA/Ca, 5% FVB/N and 17% CD1 (Fig. 1A) and results for this stage are summarised in Figs. 2–6 and Table 1. (The four genotypes are colour-coded in each figure to help distinguish them).

**Table 1:** Summary of adult eye morphology frequency results at different stages of the study.

Genotype	Morphology			Total	% normal	% severe	*P*-value†
	Normal	Mild	Severe				
**Stage 1 crosses**							
*Le-Cre^+/+^; Pax6^+/+^*	6	0	0	6	100	0	N/A
*Le-Cre^+/+^; Pax6^fl/+^*	6	0	0	6	100	0	N/A
*Le-Cre^Tg/-^; Pax6^+/+^*	4	2††	0	6	67†	0	1.0000
*Le-Cre^Tg/-^; Pax6^fl/+^*	0	2	6	8	0	75	0.0007***
**Stage 3 crosses**							
*Le-Cre^+/+^; Pax6^+/+^*	6	0	0	6	100	0	N/A
*Le-Cre^+/+^; Pax6^fl/+^*	6	0	0	6	100	0	N/A
*Le-Cre^Tg/-^; Pax6^+/+^*	2	1	3	6	33	50	0.0245*
*Le-Cre^Tg/-^; Pax6^fl/+^*	0	3	3	6	0	50	0.0245*
**Stage 4 crosses**							
*Le-Cre^+/+^; Pax6^+/+^*	4	0	0	4	100	0	N/A
*Le-Cre^+/+^; Pax6^fl/+^*	4	0	0	4	100	0	N/A
*Le-Cre^Tg/-^; Pax6^+/+^*	4	2	0	6	67	0	1.0000
*Le-Cre^Tg/-^; Pax6^fl/+^*	0	2	4	6	0	67	0.0150*
**PAX77 crosses**							
*Le-Cre^Tg/-^; Pax6^+/+^; PAX77^-/-^*	0	1	2	3	0	67	N/A
*Le-Cre^Tg/-^; Pax6^fl/+^; PAX77^-/-^*	0	0	3	3	0	100	N/A
*Le-Cre^Tg/-^; Pax6^+/+^; PAX77^Tg/-^*	0	3	0	3	0	0	0.4000
*Le-Cre^Tg/-^; Pax6^fl/+^; PAX77^Tg/-^*	0	3	0	3	0	0	0.1000

For morphology classification, “Mild” indicates mildly abnormal morphology and “Severe” indicates severely abnormal morphology.

† *P*-values for stage-1, stage-3 and stage-4 crosses are for Fisher's Exact tests of the proportion of severely abnormal eyes tested against the proportion in the *Le-Cre^+/+^; Pax6^+/+^* plus the *Le-Cre^+/+^; Pax6^fl/+^* control groups. For the PAX77 crosses, *P*-values are for Fisher's Exact tests of the proportion of severely abnormal eyes for *Le-Cre^Tg/-^; Pax6^+/+^; PAX77^Tg/-^* versus *Le-Cre^Tg/-^; Pax6^+/+^; PAX77^-/-^* and for *Le-Cre^Tg/-^; Pax6^fl/+^; PAX77^Tg/-^* versus *Le-Cre^Tg/-^; Pax6^fl/+^; PAX77^-/-^.*

*P<0.05;

***P<0.001; N/A =  not applicable.

†† Two *Le-Cre^Tg/-^; Pax6^+/+^* mice from stage-1 crosses had a very mild phenotype (irido-corneal angles appeared partly closed) – see Fig. 2D.

Haematoxylin and eosin staining of histological sections (one eye per mouse) showed that control eyes (*Le-Cre^−/−^; Pax6^+/+^*, *Le-Cre^−/−^; Pax6^fl/+^* and *Le-Cre^Tg/−^; Pax6^+/+^*) were morphologically normal (Figs. 2A–C), apart from two of the six *Le-Cre^Tg/−^; Pax6^+/+^* eyes examined, in which the irido-corneal angle appeared at least partly closed due to adhesion between the iris and peripheral cornea (Fig. 2D). All eight experimental *Le-Cre^Tg/−^; Pax6^fl/+^* eyes examined were morphologically abnormal but they varied in severity (Figs. 2E,F). Lenses were small, malformed, vacuolated and not always entirely contained within a capsule. Lens-corneal plugs (persistent lens stalks) were present in some cases (Fig. 5M), as described previously for both *Pax6^+/−^* [26], [38] and *Le-Cre^Tg/−^; Pax6^fl/+^* [28] mice. Retinal dysgenesis occurred in all *Le-Cre^Tg/−^; Pax6^fl/+^* samples examined and varied from mild folding or swirling to more severe abnormalities. In most cases, there was no pupil and pigmented tissue adhered to the corneal endothelium, which may have been a persistent, pigmented pupillary membrane, as reported for *Pax6^Leca4/+^* heterozygotes [39]. There were also irido-lenticular and irido-corneal adhesions and in some cases the irido-corneal angle appeared closed and the ciliary body appeared abnormal or hypoplastic (Fig. 2E). For frequency comparisons, eye morphology was classified as normal, mildly abnormal or severely abnormal (Table 1). For these stage-1 crosses, the frequency of eyes with severely abnormal eyes was significantly greater than in the pooled group of *Le-Cre^+/+^; Pax6^+/+^* and *Le-Cre^+/+^; Pax6^fl/+^* controls without the *Le-Cre* transgene (0/12) by Fisher's Exact test for *Le-Cre^Tg/−^; Pax6^fl/+^* eyes (6/8; *P* = 0.0007) but not for *Le-Cre^Tg/−^; Pax6^+/+^* eyes (0/6; *P* = 1.0000).

In stage 1, *Le-Cre^Tg/−^; Pax6^fl/+^* eye size varied and some were significantly smaller than the three control genotypes, which were all similar, as shown for eye mass in Figs. 3A,D,G. For some *Le-Cre^Tg/−^; Pax6^fl/+^* mice, the mass of left and right eyes differed markedly, implying that both genetic and stochastic differences can affect eye size. Comparisons of eye diameter and corneal diameter measurements (Figs. S1 and S2) showed the same trends as eye mass.

### Abnormalities and immunohistochemistry of *Le-Cre^Tg/−^; Pax6^fl/+^* corneas (stage-1 crosses)

The original focus of this study was the corneal epithelium, which was thinner in the experimental *Le-Cre^Tg/−^; Pax6^fl/+^* group than the three control groups at stage-1 (Figs. 4A–D) and this is shown quantitatively in Fig. 6. As reported for *Pax6^+/Sey-Neu^* heterozygotes [19], ectopic goblet cells were identified by PAS staining in the corneal epithelium of two of the six *Le-Cre^Tg/−^; Pax6^fl/+^* eyes examined (Fig. 4L) but none were seen in 18 control eyes (six for each genotype; Figs. 4I–K). The corneal epithelium of all four genotypes stained positively for Pax6 (Figs. 4E–H) and keratin 5 (K5; Figs. 5A–H). Staining of keratin 19 (K19), which is normally expressed in the mouse conjunctiva and limbus but not in the central cornea [40], was detected in conjunctival-limbal region of all four groups (Figs. 5N–Q). As expected, it was absent from the central cornea of the three control groups (Figs. 5I–K) but patchy staining occurred in the central corneal epithelium of the experimental *Le-Cre^Tg/−^; Pax6^fl/+^* mice (Figs. 5L,M), as reported previously for *Pax6^+/Sey-Neu^* heterozygotes [41], suggesting that the corneo-limbal boundary may be indistinct. The presence of K19 staining and goblet cells in the central corneal epithelium is consistent with either conjuctivalisation or abnormal differentiation of the *Le-Cre^Tg/−^; Pax6^fl/+^* corneal epithelium. As expected, keratin 12 (K12) immunostaining was detected throughout the corneal epithelium of all three controls but, unexpectedly, was completely absent from the corneal epithelium of all six *Le-Cre^Tg/−^; Pax6^fl/+^* eyes examined (Figs. 5R–Y). This is a significantly more severe phenotype than reported for *Pax6^+/Sey-Neu^* heterozygotes [19] [22]. Moreover, the presence of abnormalities (closure of irido-corneal angles) in some *Le-Cre^Tg/−^; Pax6^+/+^* control eyes suggested that depletion of Pax6 in the surface ectoderm, by *loxP* recombination, might not be the sole cause of eye abnormalities in the experimental *Le-Cre^Tg/−^; Pax6^fl/+^* mice.

### Corneal wound healing in *Le-Cre^Tg/−^; Pax6^fl/+^* eyes (stage-2 crosses)

Ex-vivo corneal epithelial wound healing, in the four groups, produced by *Le-Cre^Tg/−^*; *Pax6^+/+^*× *Le-Cre^−/−^*; *Pax6^fl/+^* crosses, was compared to wound healing in CBA/Ca- *Pax6^+/Sey-Neu^* heterozygotes (Fig. S3). The *Le-Cre^Tg/-^*; *Pax6^+/+^*and *Le-Cre^−/−^*; *Pax6^fl/+^* mice had been crossed to CBA/Ca for more generations and the genetic background had increased from approximately 78% CBA/Ca to approximately 93% CBA/Ca (Figs. 1A,B) so this was considered to be a new stage of the study (stage 2). The trajectory of wound healing was quite variable within groups and some CBA/Ca- *Pax6^+/Sey-Neu^* wounds increased in size during the first 6 hours, suggesting greater corneal epithelial fragility. Comparisons of the frequencies of wounds that healed within 24 hours are shown both separately for left and right eyes and for pooled samples of left and right eyes in Table S2. In each case there is a trend for fewer wounds to close in CBA/Ca- *Pax6^+/Sey-Neu^* positive controls and *Le-Cre^Tg/−^; Pax6^fl/+^* experimental mice than in *Le-Cre^−/−^; Pax6^+/+^*, *Le-Cre^−/−^; Pax6^fl/+^* or *Le-Cre^Tg/−^; Pax6^+/+^* control groups but most differences failed to reach significance by Fisher's Exact tests unless results for left and right eyes were pooled. Wound healing abnormalities in *Pax6^+/Sey-Neu^* mice have been attributed to reduced Pax6 levels [23], [24]. As wound-healing was similar in *Pax6^+/Sey-Neu^* and *Le-Cre^Tg/−^; Pax6^fl/+^* mice, the wound-healing abnormalities in *Pax6^+/Sey-Neu^* mice are likely to be at least partly mediated intrinsically via the surface ectoderm lineage.

### Morphology of experimental *Le-Cre^Tg/−^; Pax6^fl/+^* eyes is abnormal by E16.5 (stage 3 crosses)

**Figure 7:**
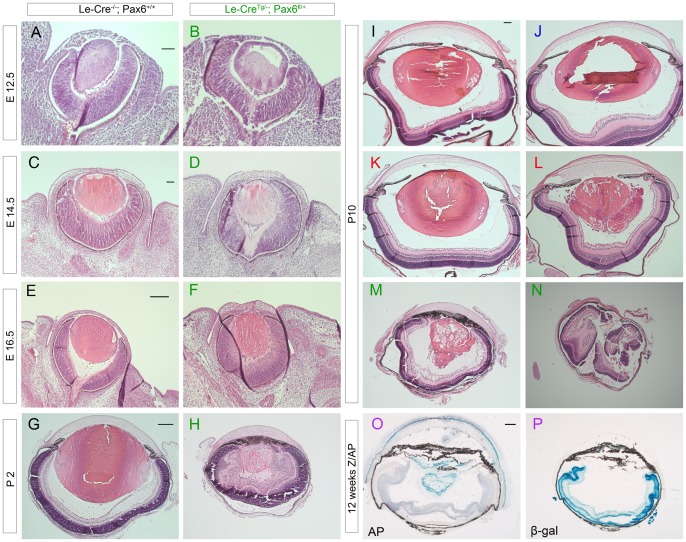
Morphology of fetal and juvenile eyes and Z/AP reporter expression in adult eyes from mice produced in stage 3. (**A–H**) Comparison of morphology in H & E stained histological sections of control *Le-Cre^−/−^; Pax6^+/+^* and experimental *Le-Cre^Tg/−^; Pax6^fl/+^* fetal and neonatal eyes from (*Le-Cre^Tg/−^*; *Pax6^+/+^* × *Le-Cre^−/−^*; *Pax6^fl/+^*) stage-3 crosses at E12.5 to P2 showing lens abnormalities in *Le-Cre^Tg/−^; Pax6^fl/+^* eyes at E16.5 (F) and more extensive ocular abnormalities in *Le-Cre^Tg/−^; Pax6^fl/+^* eyes at P2 (H). (**I–N**) Comparison of morphology in H & E stained histological sections of all three control genotypes from (*Le-Cre^Tg/−^*; *Pax6^+/+^* × *Le-Cre^−/−^*; *Pax6^fl/+^*) stage-3 crosses at P10 showing normal morphology of (I) *Le-Cre^−/−^; Pax6^+/+^*, (J) *Le-Cre^−/−^; Pax6^fl/+^* and (K) one *Le*-*Cre^Tg/−^; Pax6^+/+^* controls but abnormal morphology in (L) another *Le*-*Cre^Tg/−^; Pax6^+/+^* control and (M,N) eyes from *Le-Cre^Tg/−^; Pax6^fl/+^* experimental mice in stage 3 of this study. The lenses in I-K are normal but the lens shown in (J) was damaged during sectioning. (**O,P**) Histochemical staining for (O) placental alkaline phosphatase (blue endpoint) and (P) β-galactosidase (blue endpoint) in different sections from the same *Le-Cre^Tg/−^*; *Z/AP* adult reporter eye showing AP staining is restricted to the surface ectoderm derivatives (lens and corneal epithelium). Eye morphology in the *Le-Cre^Tg/−^*; *Z/AP* eyes was often abnormal in stage 3 (O,P), as it was for some *Le*-*Cre^Tg/−^; Pax6^+/+^* controls in stage 3 (L and Fig. 8). Scale bars: E12.5 and E14.5 (A–D)  = 50 µm; E16.5 and P2 (E–H)  = 200 µm, P10 (I–N)  = 100 µm; 12 weeks (O,P)  = 200 µm. *Abbreviations:* AP, alkaline phosphatase staining; β-gal  =  β-galactosidase staining. Colour code of panel lettering: black  =  *Le-Cre^−/−^; Pax6^+/+^*; blue  =  *Le-Cre^−/−^; Pax6^fl/+^*; red  =  *Le-Cre^Tg/−^; Pax6^+/+^*; green  =  *Le-Cre^Tg/−^; Pax6^fl/+^*; purple  =  *Le-Cre^Tg/−^*; *Z/AP.*

We next compared eye morphology (one eye per mouse) in a developmental series of H & E stained sections of fetal and juvenile control *Le-Cre^−/−^; Pax6^+/+^* and experimental *Le-Cre^Tg/−^; Pax6^fl/+^* eyes (Fig. 7) to identify when the abnormalities seen in adult *Le-Cre^Tg/−^; Pax6^fl/+^* experimental mice (Fig. 2) arose. By this stage, *Le-Cre^Tg/−^*; *Pax6^+/+^*and *Le-Cre^−/−^*; *Pax6^fl/+^* mice had been crossed to CBA/Ca for more generations and the genetic background had increased from approximately 93% to approximately 98% CBA/Ca (Figs. 1B,C) so this was considered to be stage 3. Abnormalities were first detected as disorganised lenses in E16.5 *Le-Cre^Tg/−^; Pax6^fl/+^* eyes (Fig. 7F) whereas *Le-Cre^−/−^; Pax6^+/+^* control eyes were normal (Fig. 7E). By P2 abnormalities in *Le-Cre^Tg/−^; Pax6^fl/+^* eyes were very obvious. Eyes and lenses were smaller than in controls, the retina was dysplastic (retinal swirls) and pigmented iris like-tissue adhered to the corneal endothelium (Fig. 7H).

### Eye morphology is abnormal in *Le-Cre^Tg/−^; Pax6^+/+^* controls as well as *Le-Cre^Tg/−^; Pax6^fl/+^* experimental mice from stage-3 crosses

At P10 we also examined the other two control groups (*Le-Cre^−/−^; Pax6^fl/+^* and *Le-Cre^Tg/−^; Pax6^+/+^*). As expected, all five of the experimental *Le-Cre^Tg/−^; Pax6^fl/+^* eyes examined were abnormal (Figs. 7M,N). More surprisingly, some P10 control *Le-Cre^Tg/−^; Pax6^+/+^* eyes from stage-3 crosses also displayed a much more obvious abnormal phenotype than the partly closed irido-corneal angles seen in stage 1 adults. Three of the five *Le-Cre^Tg/−^; Pax6^+/+^* control eyes examined appeared normal (Fig. 7K) but two had abnormal lenses (Fig. 7L). In contrast, all the *Le-Cre^−/−^; Pax6^+/+^* and *Le-Cre^−/−^; Pax6^fl/+^* controls examined (4 eyes per genotype) appeared normal (Figs. 7I,J).

**Figure 8:**
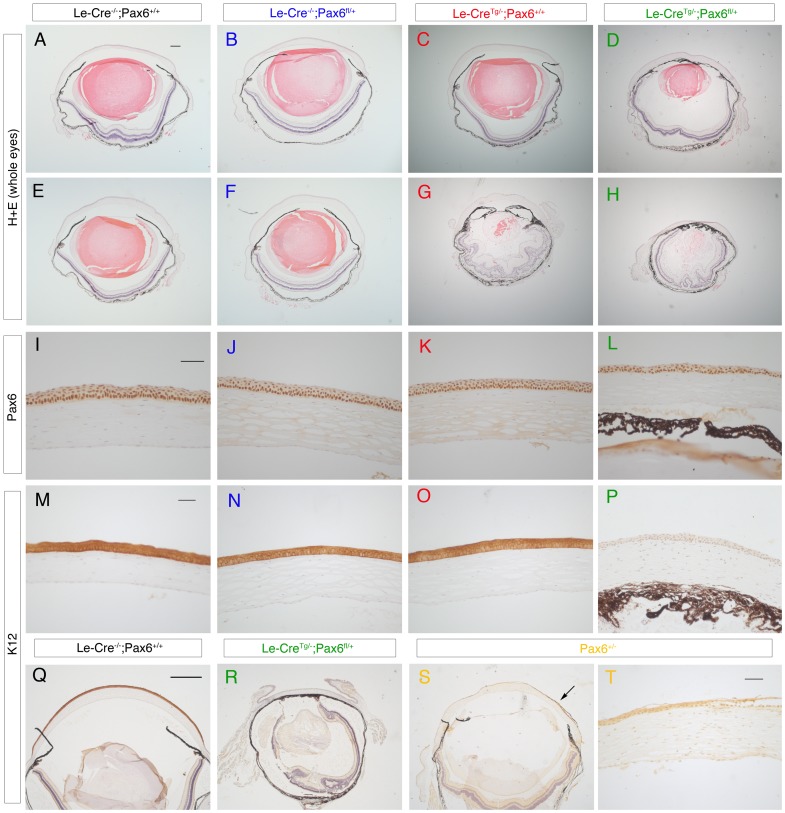
Morphology of eyes and corneal immunostaining for Pax6 and K12 from adults in stage 3. (**A–H**) H & E stained sections of adult eyes from stage-3 crosses (with a predominantly CBA/Ca genetic background) showing normal morphology of (A,E) *Le-Cre^−/−^; Pax6^+/+^*, (B,F) *Le-Cre^−/−^; Pax6^fl/+^* and (C) an *Le-Cre^Tg/−^; Pax6^+/+^* control eye but abnormal morphology of (G) another *Le-Cre^Tg/−^; Pax6^+/+^* control eye and a range of morphological abnormalities in (D,H) experimental *Le-Cre^Tg/−^; Pax6^fl/+^*eyes. (**I–L**) Pax6 immunostaining (brown endpoint) in the corneal epithelium of all four genotypes. (**M–P**) K12 immunostaining (brown endpoint) in the central corneal epithelium shows K12 is present in the corneal epithelium of all three control eyes but absent from the *Le-Cre^Tg/−^; Pax6^fl/+^* corneal epithelium. (**Q–S**) Low power views of K12 immunostaining (brown endpoint) across the whole corneal epithelium showing (Q) strong staining in an *Le-Cre^−/−^; Pax6^+/+^*control eye, (R) no staining in an *Le-Cre^Tg/−^; Pax6^fl/+^* eye and (S) weak and patchy staining in a heterozygous *Pax6^+/Sey-Neu^* eye (arrow shows a K12-positive region). (**T**) Higher power view of a K12-positive region in the corneal epithelium of an adult *Pax6^+/Sey-Neu^* heterozygote. Scale bars: A–P (shown in the first panel of each row) and T = 200 µm, Q–S (shown in Q)  = 500 µm.

The eyes of the adult control *Le-Cre^Tg/−^; Pax6^+/+^* mice from stage-3 crosses, as well as the experimental *Le-Cre^Tg/−^; Pax6^fl/+^* mice, varied in size and many were smaller than the *Le-Cre^−/−^; Pax6^+/+^* and *Le-Cre^−/−^; Pax6^fl/+^* controls, as shown by the quantitative comparisons of eye mass (Figs. 3B,E,H) plus comparisons of eye and corneal diameters (Figs. S1 and S2). Furthermore, by this stage many of the *Le-Cre^Tg/−^*; *Pax6^+/+^* stock mice, produced by crossing *Le-Cre^Tg/−^*; *Pax6^+/+^* to CBA/Ca (rather than crossing them to *Le-Cre^−/−^*; *Pax6^fl/+^* to produce mice for analysis), also had overtly small eyes (data not shown). While adult stage-3 *Le-Cre^−/−^; Pax6^+/+^* and *Le-Cre^−/−^; Pax6^fl/+^* controls continued to display a normal wild-type histological phenotype (Figs. 8A,B,E,F), four of the six *Le-Cre^Tg/−^; Pax6^+/+^* control eyes examined were morphologically abnormal (Fig. 8G) and the other two appeared normal (Fig. 8C). The types of abnormalities seen in the stage-3 *Le-Cre^Tg/−^*; *Pax6^+/+^* control mice was similar to those reported for some heterozygous *Pax6^+/−^* mice (e.g. small disorganised lens, thinner corneal epithelium, irido-corneal adhesions and irido-lenticular adhesions). The experimental *Le-Cre^Tg/−^; Pax6^fl/+^* eyes displayed a range of severe abnormalities (Figs. 8D,H) that were similar to those seen in stage-1 experimental *Le-Cre^Tg/−^; Pax6^fl/+^* eyes (Figs. 2E,F).

For stage-3 crosses, the frequency of eyes with severe abnormalities was significantly greater than in the two control groups without the *Le-Cre* transgene (0/12) by Fisher's Exact test for both *Le-Cre^Tg/−^; Pax6^fl/+^* and *Le-Cre^Tg/−^; Pax6^+/+^* eyes (3/6; *P* = 0.0245 in both cases) as shown in Table 1. Although the frequency of *Le-Cre^Tg/−^; Pax6^+/+^* eye abnormalities was higher at stage 3 than stage 1 this difference did not reach statistical significance either for all abnormalities (4/6 vs. 2/6; *P* = 0.5671) or severe abnormalities (3/6 vs. 0/6; *P* = 0.1818). However, as noted above, eye abnormalities were significantly more frequent in *Le-Cre^Tg/−^; Pax6^+/+^* than the two control groups without the *Le-Cre* transgene at stage 3 (4/6 vs. 0/12; *P* = 0.0049 for all abnormalities and 3/6 vs. 0/12; *P* = 0.0245 for just the severe abnormalities) but not at stage 1 (2/6 vs. 0/12; *P* = 0.0980 for all abnormalities and 0/6 vs. 0/12; *P* = 1.0000 for severe abnormalities). This comparison indicates that the trend for more *Le-Cre^Tg/−^; Pax6^+/+^* eye abnormalities at stage 3 than stage 1 is significant.

As in the stage-1 cross, Pax6 immunostaining occurred in the corneal epithelium of all four genotypes from the stage-3 crosses (Figs. 8I–L). K12 immunostaining was examined in six eyes from each of the four genotypes. K12 was detected throughout the corneal epithelium of all three controls (Figs. 8M–O), including the four *Le-Cre^Tg/−^; Pax6^+/+^* eyes that were morphologically abnormal, but it was again undetectable in the corneal epithelium of all the experimental *Le-Cre^Tg/−^; Pax6^fl/+^* eyes examined (Fig. 8P). Figs. 8S,T show weak and patchy K12 immunostaining in the corneal epithelium of an adult *Pax6^+/Sey-Neu^* mouse (17-weeks old) that was congenic on a CBA/Ca genetic background (>20 generations of backcrosses). This differs from both the strong staining in *Le-Cre^−/−^; Pax6^+/+^* controls (Figs. 8M,Q) and the undetectable staining of *Le-Cre^Tg/−^; Pax6^fl/+^* eyes (Figs. 8P,R) from stage-3 crosses, with a similar genetic background.

To check whether the *Le-Cre* transgene was being ectopically expressed we stained separate sections of eyes from *Le-Cre^Tg/−^; Z/AP* reporter mice for alkaline phosphatase and β-galactosidase expression. These mice had a similar genetic background to other stage 3 mice (estimated as approximately 97% CBA/Ca, 0.3% FVB/N and 2.3% CD1; Fig. 1E). The Z/AP reporter mouse uses a double reporter system to provide an assay for Cre-mediated recombination of *loxP* sites. Before recombination, cells express *lacZ* but upon recombination the *lacZ* reporter is excised and *lacZ* expression is replaced with alkaline phosphatase (AP) expression [36]. Alkaline phosphatase was expressed in the corneal epithelium and lens of *Le-Cre^Tg/−^; Z/AP* eyes (Fig. 7O), confirming that Cre-recombinase was expressed appropriately. These stage-3 *Le-Cre^Tg/−^; Z/AP* eyes had abnormal lenses and AP-positive cells were not all contained within a lens capsule. β-galactosidase staining on sections of the same eyes revealed *lacZ* expression in the retina where, as expected, no Cre-mediated excision had occurred (Fig. 7P). Thus, despite the morphological abnormalities there was no evidence for ectopic *Le-Cre* expression in these stage-3, *Le-Cre^Tg/−^; Z/AP* reporter mice.

### Eye morphology in *Le-Cre^Tg/−^; Pax6^+/+^* control eyes is improved by changing the genetic background (stage-4 crosses)

**Figure 9:**
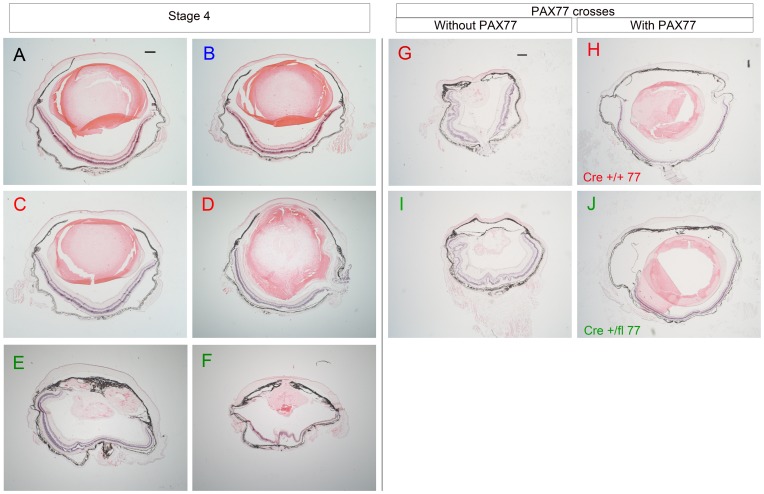
Partial rescue of adult eye abnormalities by changing the genetic background or increasing Pax6 levels. (**A–F**) H & E stained sections of adult eyes from stage 4 crosses (crossed to CBA/Ca for 8 generations and then FVB/N for 2 generations) showing normal morphology of (A) *Le-Cre^−/−^; Pax6^+/+^* and (B) *Le-Cre^−/−^; Pax6^fl/+^* control eyes and relatively few abnormalities in (C,D) *Le-Cre^Tg/−^; Pax6^+/+^* control eyes but more severe abnormalities in (E,F) experimental *Le-Cre^Tg/−^; Pax6^fl/+^*eyes. (**G–J**) H & E stained sections of adult eyes from crosses between stage-3 *Le-Cre^Tg/−^*; *Pax6^fl/+^* mice and CBA-*PAX77^Tg/−^* transgenic mice (with elevated Pax6 levels, congenic on a CBA/Ca genetic background). (**G,H**) The effect of additional Pax6 from the PAX77 transgene on *Le-Cre^Tg/−^; Pax6^+/+^* control genotype is shown by comparing the morphology of (G) the *Le-Cre^Tg/−^; Pax6^+/+^; PAX77^−/−^* control eye (without the *PAX77* transgene) and (H) the *Le-Cre^Tg/−^; Pax6^+/+^; PAX77^Tg/−^* eye (with the *PAX77* transgene). (**I,J**) The effect of additional Pax6 from the PAX77 transgene on *Le-Cre^Tg/−^; Pax6^fl/+^* experimental genotype is shown by comparing the morphology of (I) the *Le-Cre^Tg/−^; Pax6^fl/+^; PAX77^−/−^* control eye (without the *PAX77* transgene) and (J) the *Le-Cre^Tg/−^; Pax6^fl/+^; PAX77^Tg/−^* eye (with the *PAX77* transgene). The lenses in A–C, H & J were normal but some were damaged during sectioning. Scale bars A–J = 200 µm. *Abbreviations:* Cre +/+77 is *Le-Cre^Tg/−^; Pax6^+/+^*; *PAX77^Tg/^*; and Cre fl/+77 is *Le-Cre^Tg/−^; Pax6^fl/+^*; *PAX77^Tg/−^*. Colour code of panel lettering: black  =  *Le-Cre^−/−^; Pax6^+/+^*; blue  =  *Le-Cre^−/−^; Pax6^fl/+^*; red  =  *Le-Cre^Tg/−^; Pax6^+/+^* or *Le-Cre^Tg/−^; Pax6^+/+^*; *PAX77^Tg/−^*; green  =  *Le-Cre^Tg/−^; Pax6^fl/+^* or *Le-Cre^Tg/−^; Pax6^fl/+^*; *PAX77^Tg/−^*.

Comparisons of eye morphology in mice from crosses in stages 1 and 3 suggested that continued backcrossing to CBA/Ca was associated with a marked increase in abnormalities in control *Le-Cre^Tg/−^; Pax6^+/+^* eyes. In stage 4 of this study we, therefore, investigated whether increasing the contribution of the FVB/N genetic background, which was originally used to maintain the *Le-Cre* transgene, would reduce the level of abnormalities. *Le-Cre^Tg/−^*; *Pax6^+/+^* mice that had been backcrossed to CBA/Ca for 8 generations (99.6% CBA and 0.4% FVB) were backcrossed to FVB for a further two generations and then crossed to *Le-Cre^−/−^*; *Pax6^fl/+^* mice that had been backcrossed to CBA/Ca for 5 or 6 generations to produce four genotypes with genetic backgrounds estimated as 61% CBA, 38% FVB and 1% CD1 (Fig. 1F). This resulted in a significant improvement in morphology of control *Le-Cre^Tg/−^; Pax6^+/+^* eyes, although experimental *Le-Cre^Tg/−^; Pax6^fl/+^* eyes remained severely abnormal (Figs. 9A–F). A mildly abnormal phenotype (mild lens abnormalities and slight retinal swirling) was detected in 2 of the 6 *Le-Cre^Tg/−^; Pax6^+/+^* control eyes examined (Fig. 9D) but the other 4 eyes were normal (Fig. 9C).

For stage-4 crosses, the frequency of eyes with severely abnormal eyes was significantly greater than in the two control groups without the *Le-Cre* transgene (0/8) by Fisher's Exact test for *Le-Cre^Tg/−^; Pax6^fl/+^* eyes (4/6; *P* = 0.0150) but not for *Le-Cre^Tg/−^; Pax6^+/+^* eyes (0/6; *P* = 1.0000) as shown in Table 1. Although the frequency of *Le-Cre^Tg/−^; Pax6^+/+^* eye abnormalities was lower at stage 4 than stage 3 this difference was not significant either for all abnormalities (2/6 vs. 4/6; *P* = 0.5671) or severe abnormalities (0/6 vs. 3/6; *P* = 0.1818). However, the trend for fewer *Le-Cre^Tg/−^; Pax6^+/+^* eye abnormalities at stage 4 is supported by comparisons between *Le-Cre^Tg/−^; Pax6^+/+^* eyes and the two control groups without the *Le-Cre* transgene at stages 3 and 4. The frequency was significantly higher among *Le-Cre^Tg/−^; Pax6^+/+^* eyes at stage 3 (4/6 vs. 0/12; *P* = 0.0049 for all abnormalities and 3/6 vs. 0/12; *P* = 0.0245 for severe abnormalities) but not at stage 4 (2/6 vs. 0/8; *P* = 0.1648 for all abnormalities and 0/6 vs. 0/8; *P* = 1.000 for severe abnormalities).

The improved morphology of the control *Le-Cre^Tg/−^; Pax6^+/+^* eyes, in stage-4 crosses, was accompanied by a more normal and less variable eye size, as demonstrated by measurements of eye mass (Figs. 3C,F,I), eye diameter (Fig. S1) and corneal diameter (Fig. S2). Experimental *Le-Cre^Tg/−^; Pax6^fl/+^* eyes from this stage-4, FVB cross remained small but were less variable in size than those from stage 1 and stage 3 crosses (Fig. 3, Figs. S1 and S2). Pax6 and K12 immunostaining results (data not shown) were unchanged from results shown Figs. 4, 5 and 8 for stages 1 and 3.

### Increased *Pax6* expression provides a partial rescue of *Le-Cre^Tg/−^; Pax6^fl/+^* and *Le-Cre^Tg/−^; Pax6^+/+^* abnormal eye phenotypes on a predominantly CBA/Ca genetic background

To determine whether increased Pax6 levels could rescue the eye abnormalities seen in *Le-Cre^Tg/−^; Pax6^+/+^* controls from stage-3 crosses, we crossed *Le-Cre^Tg/−^; Pax6^fl/+^* mice with approximately 98% CBA/Ca genetic background to CBA/Ca-*PAX77^Tg/−^* mice (on an almost 100% CBA/Ca genetic background). This transgene contains the human *PAX6* gene so over-expresses Pax6 [33] and affects the eye and corneal phenotypes [42] [34]. The genetic background of the progeny was approximately 99% CBA/Ca, 0.2% FVB and 0.8% CD1 (Fig. 1G). Comparisons of eye phenotypes of control *Le-Cre^Tg/−^; Pax6^+/+^; PAX77^−/−^* (without the *PAX77* transgene) and *Le-Cre^Tg/−^; Pax6^+/+^; PAX77^Tg/−^* eyes (with the *PAX77* transgene) and comparisons of experimental *Le-Cre^Tg/−^; Pax6^fl/+^; PAX77^−/−^* and *Le-Cre^Tg/−^; Pax6^fl/+^; PAX77^Tg/−^* eyes are shown in Figs. 9G–J. As expected, the abnormal phenotypes of the experimental *Le-Cre^Tg/−^; Pax6^fl/+^* eyes (with reduced Pax6 levels in the surface ectoderm) was partially rescued by the presence of the *PAX77* transgene (which provides additional Pax6) as shown in Figs. 9G,H.

Although the frequency of severely abnormal eyes was lower for *Le-Cre^Tg/−^; Pax6^fl/+^; PAX77^Tg/−^* mice with the *PAX77* transgene (0/3) than for *Le-Cre^Tg/−^; Pax6^fl/+^; PAX77^−/−^* mice without *PAX77* (3/3) these differences did not reach significance by a 2-tailed Fisher's Exact test (*P* = 0.1000; Table 1) but were bordering on significance by a 1-tailed test (*P* = 0.05). Interestingly, the abnormal phenotypes of the control *Le-Cre^Tg/−^; Pax6^+/+^* eyes were also partially rescued by the presence of the *PAX77* transgene even though the genetic background was quite similar to that of the stage 3 crosses (Figs. 9I,J). Thus, the frequency of severely abnormal eyes was lower for *Le-Cre^Tg/−^; Pax6^+/+^; PAX77^Tg/−^* mice with the *PAX77* transgene (0/3) than for *Le-Cre^Tg/−^; Pax6^+/+^; PAX77^−/−^* mice without *PAX77* (2/3) but again the small sample size lacked statistical power and these differences did not reach significance by Fisher's Exact test (*P* = 0.4000; Table 1).

In both *Le-Cre^Tg/−^; Pax6^+/+^; PAX77^Tg/−^* and *Le-Cre^Tg/−^; Pax6^fl/+^; PAX77^Tg/−^* genotypes, the most noticeable improvement in the phenotype attributable to the presence of the *PAX77* transgene was normalisation of lens size and morphology and absence of retinal dysgenesis (swirling and folding), although irido-corneal adhesions remained and the retina appeared thinner than normal. The retinal hypoplasia might reflect elevated Pax6 levels in the neuroectoderm lineage because the *PAX77* transgene will produce a global increase in Pax6 but this will only be balanced by Pax6 depletion, caused by the *Le-Cre^Tg/−^; Pax6^fl/+^* genotype, in the surface ectoderm derivatives. Even if Pax6 levels are more normal in the surface ectoderm lineage they are likely to be abnormally high in the tissues derived from the neuroectoderm of the optic cup. The striking partial rescue of control *Le-Cre^Tg/−^; Pax6^+/+^* eye phenotypes by the *PAX77* transgene on a predominantly CBA/Ca genetic background suggests that low Pax6 levels may mediate the abnormal phenotype of control *Le-Cre^Tg/−^; Pax6^+/+^* eyes as well as the experimental *Le-Cre^Tg/−^; Pax6^fl/+^* eyes.

## Discussion

### Eye abnormalities in *Le-Cre^Tg/−^*; *Pax6^fl/+^* experimental mice from stage-1 crosses

Pax6 was expected to be reduced to heterozygous levels in only the surface ectoderm derivatives of *Le-Cre^Tg/−^*; *Pax6^fl/+^* mice, so it was predicted that the range of abnormalities would be either similar to those reported for *Pax6^+/−^* heterozygotes (if all their eye abnormalities were mediated via the surface ectoderm derivatives) or less extensive than those reported for *Pax6^+/−^* (if some *Pax6^+/−^* eye abnormalities were mediated via depletion of Pax6 in other lineages). Unexpectedly, the experimental *Le-Cre^Tg/−^*; *Pax6^fl/+^* mice appeared more abnormal, in some respects, than the eye phenotypes reported for global depletion of Pax6 in heterozygous *Pax6^+/Sey-Neu^* (*Pax6^+/−^*) mice [19], [22], as shown in Table 2. For example, K12 staining was undetectable in the *Le-Cre^Tg/−^*; *Pax6^fl/+^* corneal epithelium.

**Table 2:** Comparison of eye phenotypes in control *Le-Cre^Tg/-^*; *Pax6^+/+^*, experimental *Le-Cre^Tg/-^*; *Pax6^fl/+^* and different *Pax6* heterozygous mice.

Genotype	*Le-Cre^Tg/-^*; *Pax6^+/+^* (from stage 1 crosses)	*Le-Cre^Tg/-^*; *Pax6^fl/+^* (from stage 1 crosses)	*Pax6* ^+*/Sey-Neu*^	*Pax6^Sey/+^*	*Pax6^Leca4/+^*
Genetic background	∼78% CBA, ∼5% FVB & ∼17% CD1	∼78% CBA, ∼5% FVB & ∼17% CD1	75% CBA & 25% C57BL* or 100% CBA**	CD1 (outbred)	mixed (unspecified)
Reference	present study	present study	[19], [22], [41]	[47]	[39]
**Phenotypes**					
1. Eye size (mass or diameter)	normal (Fig. 3)	some are small (Fig. 3)	small	small	very small
2. Corneal epithelial layers	normal (Figs. 4-6)	reduced (Figs. 4-6)	reduced	reduced	reduced
3. Keratin 12 immunostaining in cornea	positive staining (Figs. 5T,X)	absent (Figs. 5U,Y)	reduced staining	ND	ND
4. Keratin 19 immunostaining in	limbus not cornea	limbus & patchy in cornea	limbus & cornea**	ND	ND
cornea	(Figs. 5K,P)	(Figs. 5L,M,Q)			
5. Goblet cells in corneal epithelium	absent (Fig. 4K)	present (Fig. 4L)	present	ND	absent
6. Blood vessels in cornea	none seen	none seen	present in some	present	present very early
7. Lens structure	normal (Figs. 2C,D)	cataracts and abnormal (Figs. 2E,F)	cataracts	cataracts	cataracts and vacuolated.
8. Lens-corneal plug in corneal epithelium (persistent lens stalk)	absent (Figs. 2,4,5)	lens-corneal plug in some corneas (Fig. 5M)	lens-corneal plug present	lens-corneal plug in some corneas	absent
9. Kerato-lenticular adhesions or strands	absent	absent	some adhesions	strands	adhesions
10. Irido-corneal adhesions (anterior synechia) or strands	mostly absent (Figs. 2C,D) but see point 11.	some adhesions (Figs. 2E,F, 5) – also see point 13	adhesions	adhesions	adhesions and strands
11. Irido-corneal angles	mostly open angles (Fig. 2C) but some appear partly closed (Figs. 2D, 5G,X)	Most eyes severely abnormal and angles closed (Figs. 2E,5)	closed angles	closed angles	closed angles
12. Irido-lenticular adhesions (posterior synechia)	absent (Figs. 2C,D)	some adhesions present (Fig. 2F) – also see point 13	absent	adhesions present	adhesions present
13. Iris	normal (Figs. 2C,D)	malformed iris & pigmented pupillary membrane (Figs. 2F, 5)	hypoplastic	hypoplastic	malformed iris & pigmented pupillary membrane
14. Ciliary body	normal (Figs. 2C,D)	sometimes abnormal or hypoplastic (Fig. 2E)	normal	hypoplastic	malformed
15. Retina	normal (Figs. 2C,D)	dysplastic (Figs. 2E,F)	normal	dysplastic	dysplastic

*C57BL is derived from the UK C57BL/GrFa strain via C57BL/Ola and is closely related to the C57BL/6 strain [69].

**Genetic background for *Pax6^+/Sey-Neu^* K19 study was 100% CBA/Ca [41]. ND, not done.

This striking and unexpected result was seen both at stage 1 (∼78.1% CBA/Ca) and stage 3 (∼98.1% CBA/Ca). As far as we are aware, this has not been reported previously for any heterozygous *Pax6^+/−^* mice, regardless of the *Pax6^−^* null allele involved. It is a significantly more severe phenotype than reported for *Pax6^+/Sey-Neu^* heterozygotes, which showed patchy, weak to strong K12 staining at a similar age on a genetic background comprising 75% CBA/Ca and 25% C57BL [22]. K12 staining was also detectable in corneal epithelia of adult *Pax6^+/Sey-Neu^* heterozygotes that were congenic on a CBA/Ca genetic background (Figs. 8S,T). K12 is a marker for corneal-type epithelial differentiation [43] and is regulated by Pax6 [44], [45], [46]. The absence of K12 indicates that corneal epithelial differentiation is abnormal and implies that experimental *Le-Cre^Tg/−^; Pax6^fl/+^* corneas are more severely affected than *Pax6^+/Sey-Neu^* heterozygotes.

Some stage-1 *Le-Cre^Tg/−^*; *Pax6^fl/+^* abnormalities appear more similar to those described for *Pax6^Leca4/+^* heterozygotes [39] or *Pax6^Sey/+^* heterozygotes on an outbred CD1 background [47] (Table 2), although K12 immunohistochemistry was not evaluated in either of these studies. Even during the first stage of our study, some *Le-Cre^Tg/−^*; *Pax6^+/+^* controls appeared slightly abnormal, as they had partly closed irido-corneal angles. Thus, the greater abnormalities in *Le-Cre^Tg/−^*; *Pax6^fl/+^* eyes compared to *Pax6^+/Sey-Neu^* mice could be mediated by additional, unknown effects specific to the *Le-Cre* transgene. Conversely, corneal neovascularisation occurs in *Pax6^+/−^* mice but was not seen in the *Le-Cre^Tg/−^*; *Pax6^fl/+^* experimental mice analysed, suggesting either that it is mediated by lineages other than the surface ectoderm or the *Le-Cre^Tg/−^*; *Pax6^fl/+^* genetic background is less-permissive for this phenotype than the genetic background used for the *Pax6^+/−^* studies.

### Eye abnormalities in *Le-Cre^Tg/−^*; *Pax6^+/+^* control mice from stage-3 crosses

After further backcrosses to CBA/Ca mice, eyes of *Le-Cre^Tg/−^*; *Pax6^fl/+^* experimental mice from stage 3 crosses were again severely affected but now some *Le-Cre^Tg/−^*; *Pax6^+/+^* controls also had small and abnormal eyes. The types of abnormalities seen in these *Le-Cre^Tg/−^*; *Pax6^+/+^* control mice were similar to those reported for some heterozygous *Pax6^+/−^* mice. We attribute these abnormalities to the presence of the *Le-Cre* transgene because no abnormalities were seen in the *Le-Cre^−/−^*; *Pax6^fl/+^* or *Le-Cre^−/−^*; *Pax6^+/+^* controls without the *Le-Cre* transgene. We also observed some variability in eye mass between left and right eyes from the same *Le-Cre^Tg/−^*; *Pax6^+/+^* mice, indicating that stochastic variability occurs as well as variability attributed to differences in genetic background. Liu *et al.* [5] reported variation for lens abnormalities in *Le-Cre; Six3^fl/−^* mice, which they attributed to variability in the timing and extent of *Le-Cre* activity on the NMRI genetic background that was used. However, stochastic variation in eye size has also been reported for mice with reduced or elevated Pax6 levels, which is not controlled by Cre-recombinase [34], [39].

### Reducing *Le-Cre* eye abnormalities by manipulating the genetic background or Pax6 levels

When *Le-Cre^Tg/−^*; *Pax6^+/+^* mice from generation 8 were crossed to FVB/N for two generations before crossing them to *Le-Cre^−/−^*; *Pax6^fl/+^* mice (stage 4 experiments), the eye abnormalities in *Le-Cre^Tg/−^*; *Pax6^+/+^* control progeny were less frequent and much less severe than in stage 3. This partial rescue of the eye phenotype implies that a genetic background effect is involved and the abnormal stage-3 *Le-Cre^−/−^*; *Pax6^fl/+^* phenotype is not caused by an inherited mutation. Together these results suggest that the eye abnormalities are mediated by an interaction between the *Le-Cre^Tg/−^* transgene and alleles of unknown modifier genes present in certain genetic backgrounds, including CBA/Ca. Finally, introduction of the *PAX77* transgene to the *Le-Cre^Tg/−^*; *Pax6^+/+^* genotype also reduced the severity of the eye abnormalities suggesting that increasing the Pax6 level could counteract the effects of the *Le-Cre^Tg/−^* transgene on a largely CBA/Ca genetic background.

### Abnormalities in other Cre-*loxP* experiments

A number of possible pitfalls with Cre*-loxP* experiments have been identified and reviewed elsewhere [48], [49], [50]. These include (i) unexpected Cre expression patterns in somatic tissues; (ii) unexpected Cre expression in the germ line leading to deletion of floxed genes in all cells of individuals in the next generation [51] [52]; (iii) variation among floxed alleles in their sensitivity to Cre-mediated recombination and (iv) toxic effects of Cre. The genetic background may affect the pattern of loxP recombination [53] but it is not known whether this acts by altering Cre-recombinase expression (i) or another mechanism. Collectively, these problems indicate that *Cre-loxP* experiments are not always as robust as is often assumed. In our experiments, the abnormalities occurred in *Le-Cre^Tg/−^*; *Pax6^+/+^* but not *Le-Cre^−/−^*; *Pax6^fl/+^* controls, implicating the *Le-Cre* transgene rather than *loxP*.

One type of Cre toxicity involves the action of Cre recombinase on cryptic pseudo-*loxP* sites, which are present in the endogenous mouse genome [54], [55] but, to have any detectable effect, this may require prolonged expression of high levels of Cre recombinase [56], [57], [58]. Such action may result in mutations and chromosomal abnormalities, by introducing single-stranded and double-stranded DNA breaks even if Cre-mediated recombination does not occur [55], [59]. This has been implicated as the cause of sterility in mice expressing Cre recombinase in spermatids [60]. In somatic cells a common phenotypic effect is likely to be reduced cell proliferation and cell cycle arrest [57], [61] leading to increased cell death. This may be relatively well tolerated and it has been suggested that many Cre-expressing transgenic strains that appear superficially normal are not actually completely normal, as often the effects of Cre cytotoxicity would be ameliorated by a combination of developmental selection and adaptation [48]. Nevertheless, abnormal phenotypes have been reported as a result of Cre expression in somatic cells, including (i) brain damage (e.g. reduced neuronal proliferation, increased aneuploidy and apoptosis, microencephaly and hydrocephaly) [61], [62], [63], (ii) damage to the retinal pigment epithelium [64], (iii) glucose intolerance [65], (iv) cardiomyopathy leading to heart failure [66] and (v) tetraploidy among epidermal keratinocytes [59]. Activation of CreER^T2^ transgenes with tamoxifen treatment has also been associated with abnormalities in transgenic mice, including reduced proliferation and increased apoptosis in haematopoietic tissues [63], [67]. There was no clear evidence of a genetic background effect in these studies although this possibility was considered for glucose intolerance, which was observed on two different genetic backgrounds [65].

### Possible causes of eye abnormalities in *Le-Cre^Tg/−^*; *Pax6^+/+^* control mice

The examples discussed above are thought to arise by action of Cre recombinase on endogenous pseudo-*loxP* sites. However, the eye abnormalities reported here for *Le-Cre^Tg/−^*; *Pax6^+/+^* mice are phenotypically very similar to those seen in *Pax6^+/−^* heterozygotes [19], [22], which suggests that a different mechanism may be involved. The observed reduction in *Le-Cre^Tg/−^*; *Pax6^+/+^* abnormalities seen after crossing *Le-Cre^Tg/−^* to FVB for two generations makes it unlikely that the abnormal phenotype is caused by permanent DNA damage of the sort expected for action of Cre recombinase on off-target, pseudo-*loxP* sites. Furthermore, the amelioration of the eye abnormalities in *Le-Cre^Tg/−^*; *Pax6^+/+^* mice by crossing to CBA/Ca-*PAX77^Tg/−^* suggests that the mechanism may act by affecting the dose of Pax6.

We have not investigated the mechanism underlying the *Le-Cre^Tg/−^*; *Pax6^+/+^* eye abnormalities directly but the observation that the eyes of *Le-Cre^Tg/−^*; *Pax6^+/+^*; *PAX77^Tg/−^* mice (produced by crosses of *Le-Cre^Tg/−^*; *Pax6^fl/+^* with CBA/Ca-*PAX77^Tg/−^* transgenic mice) were less severe than in *Le-Cre^Tg/−^*; *Pax6^+/+^*; *PAX77^−/−^* mice (without the *PAX77* transgene) are instructive. Firstly, crosses with *Le-Cre^Tg/−^*; *Pax6^fl/+^* mice, containing both *Le-Cre* and a floxed target gene, risk causing additional abnormalities if the *Le-Cre* transgene is ectopically expressed in the germline (as discussed above) but none was seen. Secondly, as the *PAX77^Tg/−^* mice were on a CBA/Ca genetic background (≥20 backcross generations), the *Le-Cre^Tg/−^*; *Pax6^+/+^*; *PAX77^Tg/−^* mice had no less CBA/Ca genome in their genetic background but should have higher Pax6 levels than stage-3 *Le-Cre^Tg/−^*; *Pax6^+/+^* control mice without the *PAX77* transgene. The results, therefore, argue that the eye abnormalities in our stage-3 *Le-Cre^Tg/−^*; *Pax6^+/+^* mice could be caused by a mechanism that involves Pax6 depletion. The *Le-Cre* integration site remains unknown and although it is possible that insertion of the *Le-Cre* transgene into the mouse genome disrupts expression of *Pax6* or a gene that affects *Pax6* expression, this seems unlikely because the genetic background effect is reversible. Also, homozygous *Le-Cre^Tg/Tg^* mice are viable, so if the transgene insertion disrupted expression of an endogenous gene it would have to specifically affect *Pax6* expression and cause eye defects without being homozygous lethal.

In our view, a more likely possibility is that expression of Cre recombinase, from the *Pax6-Le* regulatory sequences in the *Le-Cre* transgene, competes with the endogenous *Pax6* gene for co-factors required for *Pax6* gene expression, so reducing Pax6 production from the endogenous gene. This possibility also predicts that abnormalities observed in *Le-Cre^Tg/−^*; *Pax6^+/+^* and *Le-Cre^Tg/−^*; *Pax6^fl/+^* eyes are a consequence of altered *Pax6* expression in the surface ectoderm lineage. Consistent with this, *Le-Cre^Tg/−^*; *Pax6^+/+^* eye phenotypes were similar to some of the abnormalities reported for *Pax6^+/−^* heterozygotes [19], [22] and *Le-Cre^Tg/−^*; *Pax6^fl/+^* phenotypes were more severe than those in *Pax6^+/-^* mice. The types of eye abnormalities (microphthalmia plus cataracts) reported in abstract form for *Le-Cre^Tg/Tg^* homozygotes on a mixed genetic background [30] are also consistent with an effect that is mediated via altered Pax6 levels but less penetrant on FVB than some other genetic backgrounds. This mechanism would predict that use of *Pax6-Le* regulatory sequences to drive other transgenes (apart from *Pax6* itself) would have similar consequences on certain genetic backgrounds.

### Genetic background effects on eye phenotype

Differences in genetic background also affects the phenotype of *Pax6^−/−^* homozygotes [68] and *PAX77^Tg/−^* transgenics [34] and probably explains why some reported phenotypes of heterozygous *Pax6^Sey/+^* mice are more severe than others [47]. The existence of variants of unknown modifier genes in different genetic backgrounds which influence the expressivity and penetrance of abnormal ocular phenotypes has been proposed previously to explain genetic background effects in *Le-Cre^Tg/Tg^* homozygotes (reported in abstract form [30]) and also *Pax6^Sey/+^* or *PAX77^Tg/−^* mice [34]. If the genetic background modulates the level or effectiveness of Pax6, a genetic background that decreased the effective Pax6 level is likely to increase the abnormalities in *Le-Cre^Tg/Tg^*; *Pax6^+/+^*, *Le-Cre^Tg/−^*; *Pax6^+/+^* and *Pax6^+/−^* mice but would probably reduce the severity of the abnormalities in *PAX77^Tg/−^* transgenic mice, which are caused by elevated levels of Pax6.

Whatever mechanism is involved, our observation that eye abnormalities can occur in hemizygous *Le-Cre^Tg/−^* mice on some genetic backgrounds, in the absence of a floxed allele, serves as a cautionary tale for future studies with *Le-Cre* mice, almost all of which use *Le-Cre^Tg/−^* hemizygotes. Offspring with deleted floxed alleles are analysed in most *Le-Cre* experiments but these are also *Le-Cre^Tg/−^* hemizygotes and are often on mixed genetic backgrounds. Furthermore, many published Cre-*loxP* studies give incomplete details about which controls are analysed or do not include a Cre-positive control without a floxed allele. For example, a review of studies with a different Cre transgene showed that only 6/22 (27%) included a Cre-positive control without a floxed allele [65]. Some of the *Le-Cre* experiments cited in the Introduction also failed to include this type of control and/or used *Le-Cre* mice maintained on a mixed genetic background. Intriguingly, some of these studies reported ocular phenotypes for *Le-Cre*-induced conditional knockouts that appear to overlap with those seen in our *Le-Cre^Tg/−^*; *Pax6^+/+^* controls. This implies that the authors should consider whether any of these phenotypes could have been produced by the type of unexpected *Le-Cre* effects that we have described rather than by deletion of the floxed allele. In conclusion, our results highlight the importance of including all the relevant controls in Cre-*loxP* experiments.

## Supporting Information

Figure S1 **Variation in eye diameter for different genotypes on different genetic backgrounds.** (**A–F**) Diameter of left (A–C) and right (D–F) eyes of 12 week old mice from *Le-Cre^Tg/−^*; *Pax6^+/+^* and × *Le-Cre^−/−^*; *Pax6^fl/+^* crosses on different genetic backgrounds: (A,D) stage 1 crosses (B,E) stage 3 crosses (C,F) stage 4 crosses. (**G–I**) The percentage eye diameter difference, calculated for each mouse as (larger eye diameter - smaller eye diameter) ×100/(larger eye diameter). *Abbreviations*: WT +/+ is *Le-Cre^−/−^*; *Pax6^+/+^*; WT fl/+ is *Le-Cre^−/−^*; *Pax6^fl/+^*; Cre +/+, *Le-Cre^Tg/−^*; *Pax6^+/+^* and Cre fl/+ is *Le-Cre^Tg/−^*; *Pax6^fl/+^*. Results for all four genotypes were compared by non-parametric Kruskal-Wallis (KW) tests separately for each stage of the study (*P*-values are shown in the figure) and results for WT fl/+, Cre +/+ and Cre fl/+ were compared to WT +/+ by Dunn's multiple comparison post-hoc test: *P<0.05; **P<0.01; ***P<0.001.
(TIF)

Figure S2 **Variation in corneal diameter for different genotypes on different genetic backgrounds.** (**A–F**) Diameter of left (A–C) and right (D–F) corneas of 12 week old mice from *Le-Cre^Tg/−^*; *Pax6^+/+^* and × *Le-Cre^−/−^*; *Pax6^fl^* crosses on different genetic backgrounds: (A,D) stage 1 crosses (B,E) stage 3 crosses (C,F) stage 4 crosses. (**G,I**) The percentage cornea diameter difference, calculated for each mouse as (larger cornea diameter – smaller cornea diameter) ×100/(larger cornea diameter). *Abbreviations:* WT +/+ is *Le-Cre^−/−^*; *Pax6^+/+^*; WT fl/+ is *Le-Cre^−/−^*; *Pax6^fl/+^*; Cre +/+ is *Le-Cre^Tg/−^*; *Pax6^+/+^* and Cre fl/+ is *Le-Cre^Tg/−^*; *Pax6^fl/+^*. Results for all four genotypes were compared by non-parametric Kruskal-Wallis (KW) tests separately for each stage of the study (*P*-values are shown in the figure) and results for WT fl/+, Cre +/+ and Cre fl/+ were compared to WT +/+ by Dunn's multiple comparison post-hoc test: *P<0.05; **P<0.01; ***P<0.001.
(TIF)

Figure S3 **Comparison of corneal epithelial wound healing rates among different groups (from stage 2 crosses).** (**A–C**) Mean corneal epithelial wound diameters at different times for (A) left eyes, (B) right eyes and (C) both eyes for each of the five groups compared. (**D–H**) Corneal epithelial wound for individual corneas for (D) *Le-Cre^−/−^*; *Pax6^+/+^* (E) *Le-Cre^−/−^*; *Pax6^fl/+^* (F) *Le-Cre^Tg/−^*; *Pax6^+/+^* (G) *Le-Cre^Tg/−^*; *Pax6^fl/+^* and (H) *Pax6^+/Sey-Neu^*. *Abbreviations:* WT +/+ is *Le-Cre^−/−^*; *Pax6^+/+^*; WT fl/+ is *Le-Cre^−/−^*; *Pax6^fl/+^*; Cre +/+ is *Le-Cre^Tg/−^*; *Pax6^+/+^*; Cre fl/+ is *Le-Cre^Tg/−^*; *Pax6^fl/+^* and Pax6^+/*−*^ is *Pax6^+/Sey-Neu^*.
(TIF)

Table S1 **Genetic backgrounds of mice used in different stages of the study.**
(PDF)

Table S2 **Frequency of healed wounds after 24 hours.**
(PDF)

Supplementary Data S1 **Supplementary Data for** **Figs. 3** **&** **6** **and Figs. S1, S2 & S3.**
(XLSX)
