# Supplementary figures and images for: Hemizygous Le-Cre Transgenic Mice Have Severe Eye Abnormalities on Some Genetic Backgrounds in the Absence of LoxP Sites
Source: PLoS One. 2014 Oct 1;9(10):e109193. doi: 10.1371/journal.pone.0109193 (PMC4182886; doi:10.1371/journal.pone.0109193)

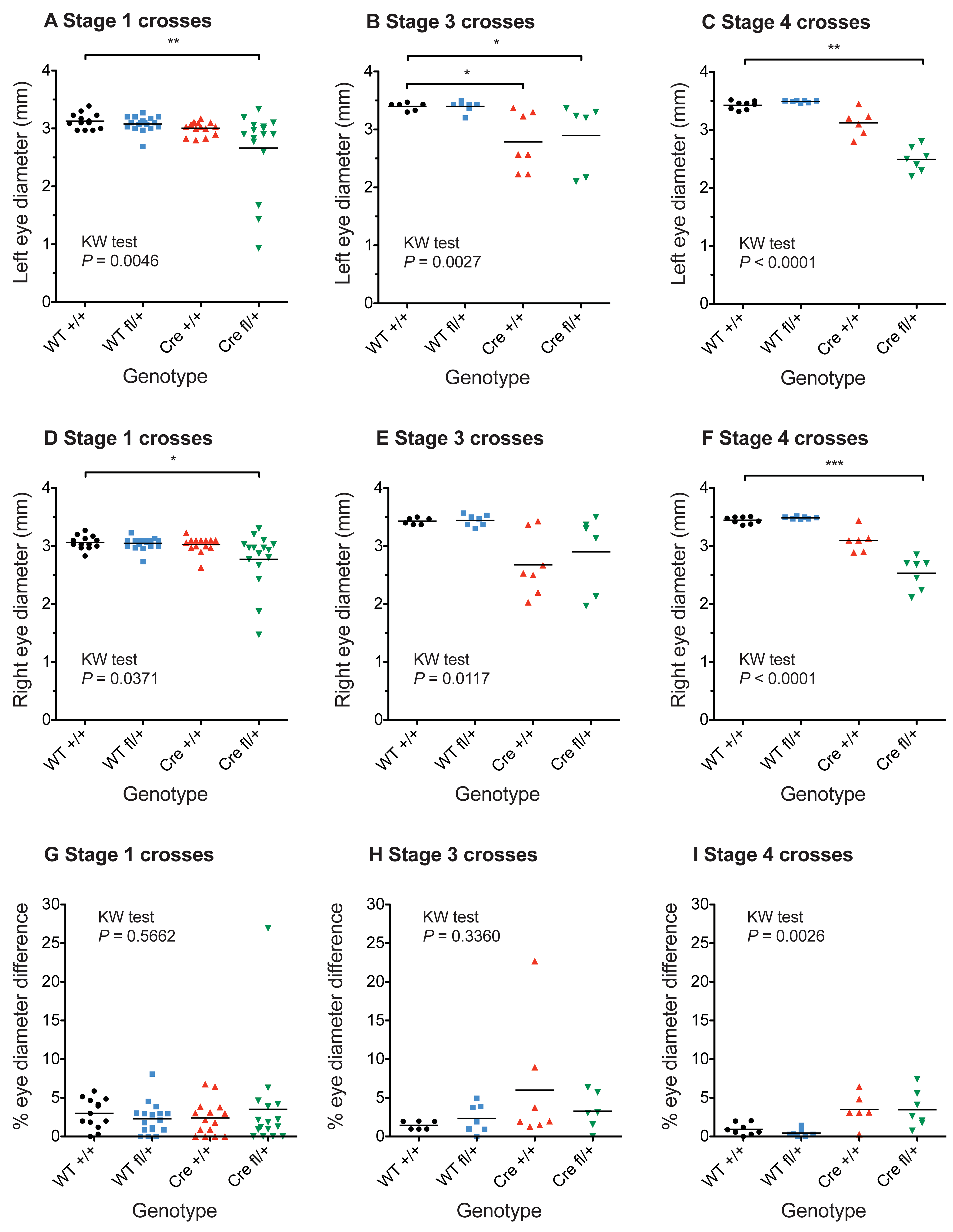

Supplement: Figure S1 — Variation in eye diameter for different genotypes on different genetic backgrounds. (A–F) Diameter of left (A–C) and right (D–F) eyes of 12 week old mice from Le-CreTg/−; Pax6+/+ and × Le-Cre−/−; Pax6fl/+ crosses on different genetic backgrounds: (A,D) stage 1 crosses (B,E) stage 3 crosses (C,F) stage 4 crosses. (G–I) The percentage eye diameter difference, calculated for each mouse as (larger eye diameter - smaller eye diameter) ×100/(larger eye diameter). Abbreviations: WT +/+ is Le-Cre−/−; Pax6+/+; WT fl/+ is Le-Cre−/−; Pax6fl/+; Cre +/+, Le-CreTg/−; Pax6+/+ and Cre fl/+ is Le-CreTg/−; Pax6fl/+. Results for all four genotypes were compared by non-parametric Kruskal-Wallis (KW) tests separately for each stage of the study (P-values are shown in the figure) and results for WT fl/+, Cre +/+ and Cre fl/+ were compared to WT +/+ by Dunn's multiple comparison post-hoc test: *P<0.05; **P<0.01; ***P<0.001. (TIF) [file pone.0109193.s001.tif]

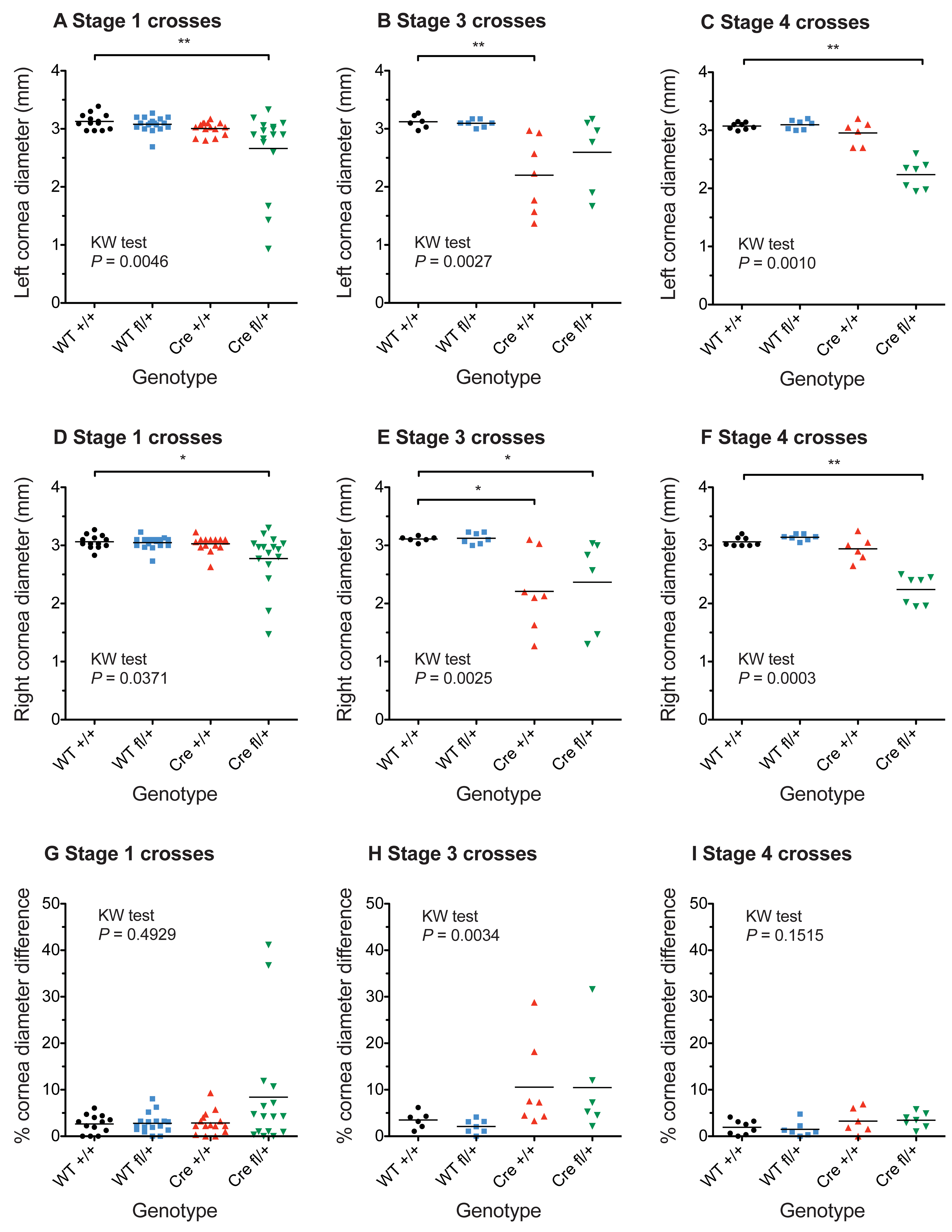

Supplement: Figure S2 — Variation in corneal diameter for different genotypes on different genetic backgrounds. (A–F) Diameter of left (A–C) and right (D–F) corneas of 12 week old mice from Le-CreTg/−; Pax6+/+ and × Le-Cre−/−; Pax6fl crosses on different genetic backgrounds: (A,D) stage 1 crosses (B,E) stage 3 crosses (C,F) stage 4 crosses. (G,I) The percentage cornea diameter difference, calculated for each mouse as (larger cornea diameter – smaller cornea diameter) ×100/(larger cornea diameter). Abbreviations: WT +/+ is Le-Cre−/−; Pax6+/+; WT fl/+ is Le-Cre−/−; Pax6fl/+; Cre +/+ is Le-CreTg/−; Pax6+/+ and Cre fl/+ is Le-CreTg/−; Pax6fl/+. Results for all four genotypes were compared by non-parametric Kruskal-Wallis (KW) tests separately for each stage of the study (P-values are shown in the figure) and results for WT fl/+, Cre +/+ and Cre fl/+ were compared to WT +/+ by Dunn's multiple comparison post-hoc test: *P<0.05; **P<0.01; ***P<0.001. (TIF) [file pone.0109193.s002.tif]

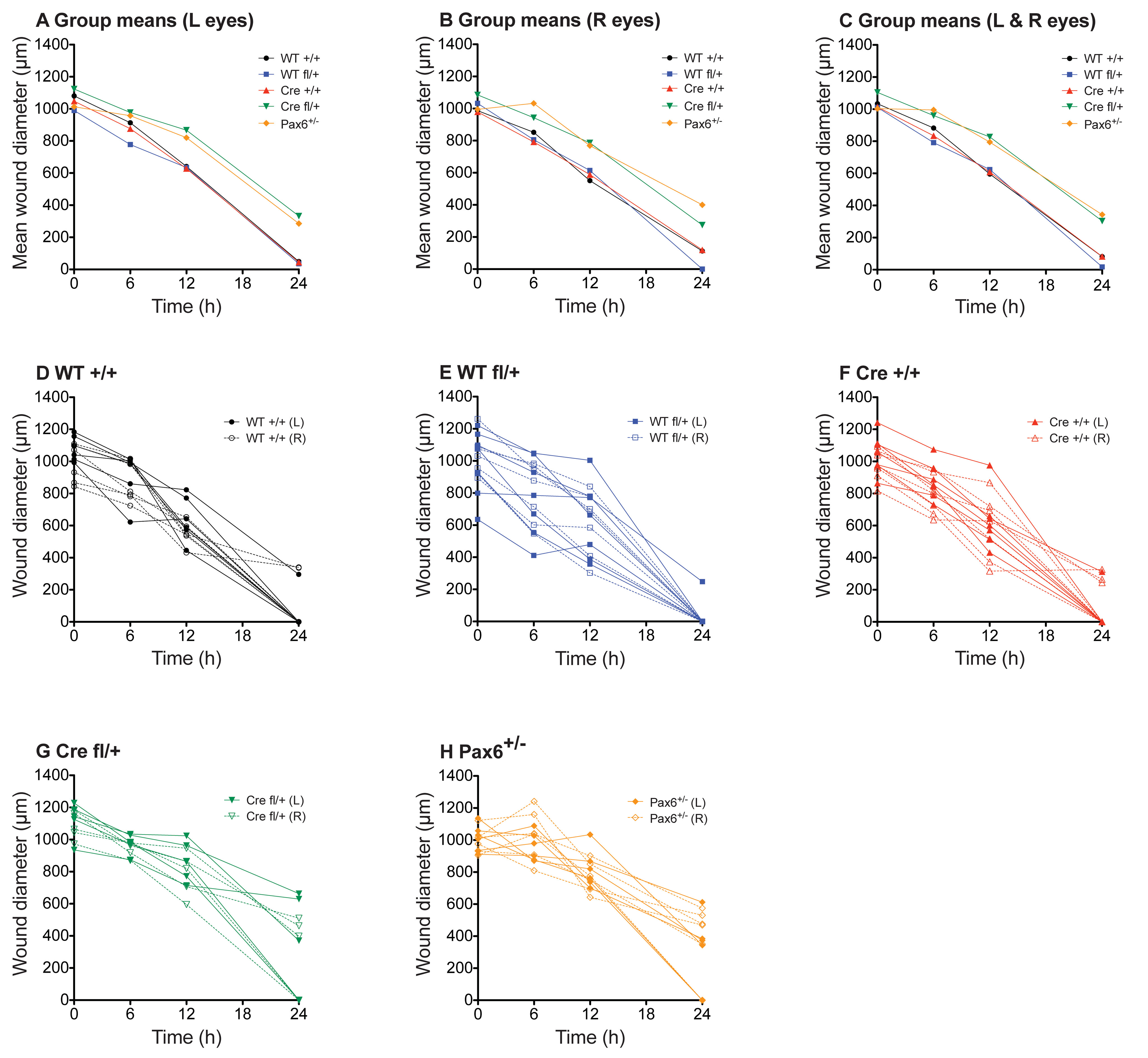

Supplement: Figure S3 — Comparison of corneal epithelial wound healing rates among different groups (from stage 2 crosses). (A–C) Mean corneal epithelial wound diameters at different times for (A) left eyes, (B) right eyes and (C) both eyes for each of the five groups compared. (D–H) Corneal epithelial wound for individual corneas for (D) Le-Cre−/−; Pax6+/+ (E) Le-Cre−/−; Pax6fl/+ (F) Le-CreTg/−; Pax6+/+ (G) Le-CreTg/−; Pax6fl/+ and (H) Pax6+/Sey-Neu. Abbreviations: WT +/+ is Le-Cre−/−; Pax6+/+; WT fl/+ is Le-Cre−/−; Pax6fl/+; Cre +/+ is Le-CreTg/−; Pax6+/+; Cre fl/+ is Le-CreTg/−; Pax6fl/+ and Pax6+/− is Pax6+/Sey-Neu. (TIF) [file pone.0109193.s003.tif]
