# Supplementary material for: Hemizygous Le-Cre Transgenic Mice Have Severe Eye Abnormalities on Some Genetic Backgrounds in the Absence of LoxP Sites
Source: PLoS One. 2014 Oct 1;9(10):e109193. doi: 10.1371/journal.pone.0109193 (PMC4182886; doi:10.1371/journal.pone.0109193)
Supplement: Table S1 — Genetic backgrounds of mice used in different stages of the study. (PDF) [file pone.0109193.s004.pdf]

Supplementary Table S1. Genetic backgrounds of mice used in different stages of the study

Supplementary Table S1A
***Le-Cre* × Floxed *Pax6* (*Le-Cre*$^{Tg/-}$;*Pax6*$^{+/+}$ × *Le-Cre*$^{-/-}$;*Pax6*$^{fl/+}$) cross – Stage 1 (adults)**

Parental stocks
Le-Cre$^{Tg/-}$ originally on the FVB/N genetic background. Crossed to CBA/Ca for 3-4 generations (N)
Pax6$^{fl/+}$ originally on the CD1 genetic background. Crossed to CBA/Ca for 1-2 generations (N)

Cross	*Le-Cre*$^{Tg/-}$ parent			*Pax6*$^{fl/+}$ parent			Offspring analysed		
	Backcrossed to CBA/Ca			Backcrossed to CBA/Ca			Likely genetic background		
	N	% CBA	% FVB	N	% CBA	% CD1	% CBA	% FVB	% CD1
Le-FP6/1	3	87.50	12.5	1	50.0	50.0	68.75	6.25	25.0
Le-FP6/2	3	87.50	12.5	2	75.0	25.0	81.25	6.25	12.5
Le-FP6/4	4	93.75	6.25	2	75.0	25.0	84.38	3.13	12.5
**Expected average genetic background**							**78.13**	**5.21**	**16.67**

Le-Cre$^{Tg/-}$ = *Le-Cre*$^{Tg/-}$;*Pax6*$^{+/+}$ genotype. *Pax6*$^{fl/+}$ = *Le-Cre*$^{-/-}$;*Pax6*$^{fl/+}$ genotype.

"Le-FP6/1" refers to cross name "Le-FP6", mating pair number 1. The offspring from mating pairs Le-FP6/1–3 were born in June - October 2010. N = number of backcross generations to CBA/Ca

Supplementary Table S1B
***Le-Cre* × Floxed *Pax6* – Stage 2 (wound healing experiment)**

Parental stocks
Le-Cre$^{Tg/-}$ originally on FVB/N genetic background. Crossed to CBA/Ca for 6 generations (N)
Pax6$^{fl/+}$ originally on CD1 genetic background. Crossed to CBA/Ca for 3 generations (N)

Cross	*Le-Cre*$^{Tg/-}$ parent			*Pax6*$^{fl/+}$ parent			Offspring analysed		
	Backcrossed to CBA/Ca			Backcrossed to CBA/Ca			Likely genetic background		
	N	% CBA	% FVB	N	% CBA	% CD1	% CBA	% FVB	% CD1
Le-FP6/7	6	98.44	1.56	3	87.50	12.50	92.97	0.78	6.25
Le-FP6/8	6	98.44	1.56	3	87.50	12.50	92.97	0.78	6.25
**Expected average genetic background**							**92.97**	**0.78**	**6.25**

The offspring from mating pairs Le-FP6/7 and 8 were born in June – September 2011.

Supplementary Table S1C
***Le-Cre* × Floxed *Pax6* – Stage 3 (E12.5 fetal to P10 juvenile)**

Parental stocks
Le-Cre$^{Tg/-}$ originally on FVB/N genetic background. Crossed to CBA/Ca for 7-8 generations (N)
Pax6$^{fl/+}$ originally on CD1 genetic background. Crossed to CBA/Ca for 5-6 generations (N)

Cross	*Le-Cre*$^{Tg/-}$ parent			*Pax6*$^{fl/+}$ parent			Offspring analysed		
	Backcrossed to CBA/Ca			Backcrossed to CBA/Ca			Likely genetic background		
	N	% CBA	% FVB	N	% CBA	% CD1	% CBA	% FVB	% CD1
TM 1	8	99.61	0.39	6	98.44	1.56	99.02	0.20	0.78
TM 2	7	99.22	0.78	5	96.88	3.13	98.05	0.39	1.56
TM 3	7	99.22	0.78	5	96.88	3.13	98.05	0.39	1.56
TM 4	8	99.61	0.39	5	96.88	3.13	98.24	0.20	1.56
TM 5	8	99.61	0.39	6	98.44	1.56	99.02	0.20	0.78
TM 6	7	99.22	0.78	5	96.88	3.13	98.05	0.39	1.56
TM 7	7	99.22	0.78	5	96.88	3.13	98.05	0.39	1.56
TM 8	8	99.61	0.39	5	96.88	3.13	98.24	0.20	1.56
TM 9	8	99.61	0.39	5	96.88	3.13	98.24	0.20	1.56
**Expected average genetic background**							**98.33**	**0.28**	**1.39**

Timed matings (TM) 1-9 to produce fetuses were set up in February – May 2012.

Supplementary Table S1D
***Le-Cre* × Floxed *Pax6* – Stage 3 (adults)**

Parental stocks
Le-Cre$^{Tg/-}$ originally on FVB/N genetic background. Crossed to CBA/Ca for 7 generations (N)
Pax6$^{fl/+}$ originally on CD1 genetic background. Crossed to CBA/Ca for 5 generations (N)

Cross	*Le-Cre*$^{Tg/-}$ parent			*Pax6*$^{fl/+}$ parent			Offspring analysed		
	Backcrossed to CBA/Ca			Backcrossed to CBA/Ca			Likely genetic background		
	N	% CBA	% FVB	N	% CBA	% CD1	% CBA	% FVB	% CD1
Le-FP6/11	7	99.22	0.78	5	96.88	3.13	98.05	0.39	1.56
Le-FP6/12	7	99.22	0.78	5	96.88	3.13	98.05	0.39	1.56
Le-FP6/13	7	99.61	0.39	5	96.88	3.13	98.24	0.20	1.56
**Expected average genetic background**							**98.11**	**0.33**	**1.56**

The offspring from mating pairs Le-FP6/11-13 were born in March – May 2012.

Supplementary Table S1E
***Le-Cre* × Z/AP reporter mice – Genetic background equivalent to Stage 3**

Parental stocks
Le-Cre$^{Tg/-}$ originally on FVB/N genetic background. Crossed to CBA/Ca for 7-8 generations (N)
Z/AP originally on CD1 genetic background. Crossed to CBA/Ca for 4-5 generations (N)

Cross	*Le-Cre*$^{Tg/-}$ parent			*Z/AP* parent			Offspring analysed		
	Backcrossed to CBA/Ca			Backcrossed to CBA/Ca			Likely genetic background		
	N	% CBA	% FVB	N	% CBA	% CD1	% CBA	% FVB	% CD1
Le-ZAP/4	7	99.22	0.78	5	96.88	3.13	98.05	0.39	1.56
Le-ZAP/5	8	99.61	0.39	4	93.75	6.25	96.68	0.20	3.13
**Expected average genetic background**							**97.36**	**0.29**	**2.34**

The offspring from mating pairs Le-ZAP/4-5 were born in December 2011 – March 2012.

Supplementary Table S1F
***Le-Cre* × Floxed *Pax6* – Stage 4 (adults from crosses to FVB/N)**

Parental stocks
Le-Cre$^{Tg/-}$ originally on FVB/N genetic background. Crossed to CBA/Ca for 8 generations followed by 2 generations of crosses to FVB/N
Pax6$^{fl/+}$ originally on CD1 genetic background. Crossed to CBA/Ca for 5-6 generations (N)

Cross	*Le-Cre*$^{Tg/-}$ parent			*Pax6*$^{fl/+}$ parent			Offspring analysed		
	Backcrossed to CBA & FVB			Backcrossed to CBA/Ca			Likely genetic background		
	N	% CBA	% FVB	N	% CBA	% CD1	% CBA	% FVB	% CD1
FLE-FP6/1	8C+2FVB	24.90	75.10	6	98.44	1.56	61.67	37.55	0.78
FLE-FP6/2	8C+2FVB	24.90	75.10	5	96.88	3.13	60.89	37.55	1.56
**Expected average genetic background**							**61.28**	**37.55**	**1.17**

The offspring from mating pairs FLE-FP6/1-2 were born in October 2012.

Supplementary Table S1G
(*Le-Cre* × Floxed *Pax6*) – adults from crosses to PAX77 mice

First Cross: Parental stocks
Le-Cre$^{Tg/-}$ originally on FVB/N genetic background. Crossed to CBA/Ca for 6 generations (N)
Pax6$^{fl/+}$ originally on CD1 genetic background. Crossed to CBA/Ca for 5 generations (N)

First Cross	*Le-Cre*$^{Tg/-}$ parent			*Pax6*$^{fl/+}$ parent			Offspring analysed		
	Backcrossed to CBA/Ca			Backcrossed to CBA/Ca			Likely genetic background		
	N	% CBA	% FVB	N	% CBA	% CD1	% CBA	% FVB	% CD1
LE-FP6/8	6	99.22	0.78	5	96.88	3.13	98.05	0.39	1.56

Second Cross: Parental stocks
Le-Cre$^{Tg/-}$;*Pax6*$^{fl/+}$ Genetic background predicted from first cross
PAX77$^{Tg/-}$ originally on CD1 genetic background. Crossed to CBA/Ca for ≥20 generations (N)

Second Cross	*Le-Cre*$^{Tg/-}$;*Pax6*$^{fl/+}$ parent			*PAX77*$^{Tg/-}$ parent			Offspring analysed		
	Genetic background			Backcrossed to CBA/Ca			Likely genetic background		
	% CBA	% FVB	% CD1	N	% CBA	% CD1	% CBA	% FVB	% CD1
LE-FP6-77/1	98.05	0.39	1.56	≥20	100	0	99.02	0.20	0.78
LE-FP6-77/2	98.05	0.39	1.56	≥20	100	0	99.02	0.20	0.78
**Expected average genetic background**							**99.02**	**0.20**	**0.78**

The offspring from mating pairs LE-FP6-77/1 and 2 were born in January – May 2012.
