# Supplementary material for: Hemizygous Le-Cre Transgenic Mice Have Severe Eye Abnormalities on Some Genetic Backgrounds in the Absence of LoxP Sites
Source: PLoS One. 2014 Oct 1;9(10):e109193. doi: 10.1371/journal.pone.0109193 (PMC4182886; doi:10.1371/journal.pone.0109193)
Supplement: Table S2 — Frequency of healed wounds after 24 hours. (PDF) [file pone.0109193.s005.pdf]

Supplementary Table S2: Frequency of healed wounds after 24 hours

Left eyes

Genotype	Healed	Not healed	Total	% healed
WT +/+	5	1	6	83
WT fl/+	6	1	7	86
Cre +/+	6	1	7	86
Cre fl/+	2	3	5	40
Pax6+/-	2	4	6	33

Fisher's Exact test *P*-values			
WT fl/+	Cre +/+	Cre fl/+	Pax6+/-
1.0000 NS	1.0000 NS	0.2424 NS	0.2424 NS
-	1.0000 NS	0.2222 NS	0.1026 NS
-	-	0.2222 NS	0.1026 NS
-	-	-	1.0000 NS
-	-	-	-

Right eyes

Genotype	Healed	Not healed	Total	% healed
WT +/+	4	2	6	67
WT fl/+	7	0	7	100
Cre +/+	4	3	7	57
Cre fl/+	2	3	5	40
Pax6+/-	1	5	6	17

Fisher's Exact test *P*- values			
WT fl/+	Cre +/+	Cre fl/+	Pax6+/-
0.1923 NS	1.0000 NS	0.5671 NS	0.2424 NS
-	0.1923 NS	0.0455*	0.0047**
-	-	1.0000 NS	0.2657 NS
-	-	-	0.5455 NS
-	-	-	-

Left and right eyes

Genotype	Healed	Not healed	Total	% healed
WT +/+	9	3	12	75
WT fl/+	13	1	14	93
Cre +/+	10	4	14	71
Cre fl/+	4	6	10	40
Pax6+/-	3	9	12	25

Fisher's Exact test *P*- values			
WT fl/+	Cre +/+	Cre fl/+	Pax6+/-
0.3061 NS	1.0000 NS	0.1920 NS	0.0391*
-	0.3259 NS	0.0088**	0.0008***
-	-	0.2112 NS	0.0472*
-	-	-	0.6517 NS
-	-	-	-

Abbreviations: WT +/+ is *Le-Cre*$^{-/-}$;*Pax6*$^{+/+}$; WT fl/+ is *Le-Cre*$^{-/-}$;*Pax6*$^{fl/+}$; Cre +/+ , *Le-Cre*$^{Tg/-}$;*Pax6*$^{+/+}$, Cre fl/+ is *Le-Cre*$^{Tg/-}$;*Pax6*$^{fl/+}$ and Pax6+/- is *Pax6*$^{+/Sey\text{-}Neu}$. **P*<0.05; ***P*<0.01; ****P*<0.001; NS = not significant.
